# The influence of the antithymocyte globulin dose on clinical outcomes of patients undergoing kidney retransplantation

**DOI:** 10.1371/journal.pone.0251384

**Published:** 2021-05-12

**Authors:** Kamilla Linhares, Julia Bernardi Taddeo, Marina Pontello Cristelli, Henrique Proença, Klaus Nunes Ficher, Renato de Marco, Maria Gerbase-DeLima, Jose Medina-Pestana, Helio Tedesco-Silva

**Affiliations:** 1 Division of Nephrology, Hospital do Rim, Universidade Federal de São Paulo, São Paulo, Brazil; 2 Immunogenetics Institute, Associação Fundo de Incentivo à Pesquisa, São Paulo, Brazil; Medical University of Gdansk, POLAND

## Abstract

Optimizing antithymocyte globulin (rATG) dosage is critical for high immunological risk patients undergoing a repeat kidney transplant. This natural retrospective cohort study compared clinical outcomes of two successive cohorts of consecutive recipients of retransplants receiving 5 x 1 mg/kg (rATG-5, n = 100) or a single 3 mg/kg (rATG-3, n = 110) dose of rATG induction therapy. All patients had negative complement-dependent cytotoxicity crossmatch and no anti-HLA A, B, DR donor-specific antibodies (DSA). The primary endpoint was efficacy failure (first biopsy-proven acute rejection, graft loss, or death) at 12 months. There was no difference in the cumulative incidence of efficacy failure (18.0% vs. 21.8%, HR = 1.22, 95% CI 0.66–2.25), respectively. There were no differences in 3-years freedom from biopsy proven acute rejection, and patient, graft, and death-censored graft survivals. There were no differences in the incidence of surgical complications (25.0% vs. 18.2%; p 0.151), early hospital readmission (27.8% vs. 29.5%; p = 0.877) and CMV infections (49% vs. 40%; p = 0.190). There were also no differences in the incidence (59.6% vs. 58.7%, p = 0.897) and duration of delayed graft function but a stable difference in estimate glomerular filtration rate was observed from month 1 (54.7±28.8 vs. 44.1±25.3 ml/min/1.73 m^2^, p = 0.005) to month 36 (51.1±27.7 vs. 42.5±24.5, p = 0.019). Mean urinary protein concentration (month 36: 0.38±0.81 vs. 0.70±2.40 g/ml, p = 0.008) and mean chronic glomerular Banff score in for cause biopsies (months 4–36: 0.0±0.0 vs. 0.04±0.26, p = 0.044) were higher in the rATG-3 group. This cohort analysis did not detect differences in the incidence of efficacy failure and in safety outcomes at 12 months among recipients of kidney retransplants without A, B, and DR DSA, receiving induction therapy with a single 3 mg/kg rATG dose or the traditional 5 mg/kg rATG.

## Introduction

The number of patients requiring repeat kidney transplants is increasing [1, 2]. According to the most recent OPTN/SRTR 2019 Annual Data Report, 16.3% (2009), 14.4% (2014), and 11.8% (2019) of the patients on the waiting list had a history of a previous transplant [3]. Traditionally, these patients are considered to have a high immunological risk for early acute rejection and graft loss [4]. Particular to this specific population is the previous sensitization to HLA antigens and possible reexposure to mismatched HLA antigens, even with a negative crossmatch [5]. Consequently, induction therapy with lymphocyte depleting agents is recommended [6]. Among several protocols, the use of 5 consecutive 1 mg/kg daily doses of rATG 5 mg/kg, adjusted based on either total or CD3 positive lymphocyte counts, is routinely used [7]. Specifically among patients undergoing kidney retransplants, a recent analysis of data from 14,336 patients extracted from the United States Renal Data System (USRDS) registry showed no significant differences between induction groups for outcomes of delayed graft function, 1-year acute rejection, 1-year BK virus or patient death [8]. More recently, reduced rATG dosing regimens have been proposed based on immunological risk stratification. Cumulative rATG doses of 3 mg/kg were administered to nonsensitized living donor recipients, 4.5 mg/kg to nonsensitized deceased donor recipients and 6 mg/kg to higher immunologic risk recipients, including those with history of prior transplant. One-year rejection rates in the first 2 groups were 8.3% and 8.8%, respectively [9].

The assessment of immunological risk has been improving with the development of new tools and better mechanistic understanding of immune reactions in sensitized patients. The routine use of solid phase assays provides detailed analysis of the presence of preformed anti-HLA antibodies, allowing for the implementation of allocation policies with reduced immunological risk [10].

On Aug 1^st^, 2009, changes in the allocation system for recipients of deceased donor kidney allografts were implemented in our center. Besides the negative complement-dependent cytotoxicity (CDC) crossmatch, all patients undergoing kidney transplantation should not have pre-formed anti-HLA A, B and DR donor specific antibodies with mean fluorescence intensity (MFI) above 1500. Inherently, this strategy is associated with a lower risk for early acute rejection and graft loss [11]. Based on this assumption, we decided to change our induction protocol by reducing the dose of rATG from 5 to a single 3 mg/kg dose since Jun 16^th^, 2014, after determination of its safety and efficacy in a randomized trial [12]. Our hypothesis was that in high immunological risk patients undergoing retransplantation without pre-formed HLA A, B, DR donor specific antibodies, the use of a single 3 mg/kg dose of rATG would provide comparable efficacy to the traditional 5 mg/kg dose, with possible safety benefits related to surgical complications, number of early hospital readmissions (EHR) and incidence of CMV infection.

## Materials and methods

### Study design

This was a sequential cohort experiment in which all kidney transplant recipients started receiving 3 mg/kg of rATG for induction therapy from June 17^th^, 2014, at our institution. Therefore, two retrospective cohorts of consecutive adult patients undergoing repeat kidney transplants were constructed. We identified all consecutive kidney retransplants who received 5 mg/kg r-ATG (rATG-5 group) from January 1^st^, 2010 to June 16^th^, 2014 and all consecutive kidney retransplants who received 3 mg/kg r-ATG (rATG-3 group) from June 17th, 2014 to October 9^th^, 2016. Data was collected up to October 31^st^, 2019. There were no further changes in the protocols or in clinical practice over the time frame of this study. We compared the incidence of acute rejection, patient and graft survivals, and the incidence of early hospital readmissions, surgical complications and CMV infection.

### Ethics

Kidney transplants were performed at the Hospital do Rim with organs recovered from deceased donors under the National and São Paulo State secretary allocation policy, and from living donors, according to the National legislation. The national regulation does not mandate registration of donors. None of the transplant donors was from a vulnerable population and all donors or next of kin provided written informed consent that was freely given. Informed consent was obtained from all donors or their next of kin, prior to organ recovery. Informed consent forms for deceased (S1 Appendix: informed consent form for solid organs and tissues donation–deceased donor–over 18 years-old) and living donors (S2 Appendix: Term of authorization for kidney donation from transplantation) are provided. According to Brazilian legislation, no medical costs or other cash payments are allowed to the family of the donor. Record data from kidney transplants performed between 01Jan2010 to 09Oct2016 were screened for this analysis. The anonymized data set necessary to replicate our study findings is provided (S3 Appendix: Master File). The research project was approved by the local Research Ethics Committee of the Federal University of São Paulo (CAAE 02285018.2.5505). The IRB waived the requirement to obtain informed consent because of the retrospective nature of the record review, the lack of interference with the rights and welfare, and the lack of risk to the participants. All data were retrieved from the electronic medical records and were fully anonymized before analysis. Medical records of the Hospital do Rim (Fundação Oswaldo Ramos) were accessed between 01/06/2017 and 01/10/2020.

### Study population

All patients in both cohorts had a negative complement dependent cytotoxicity crossmatch, no preformed A, B and DR donor specific antibodies with mean fluorescence intensity higher than 1500 in both historical and current sera and had received an ABO compatible repeat renal allograft from living or deceased donors. To identify anti-HLA antibodies in serum, flowPRA screening test was performed, followed by single antigen bead assay to identify the specificity of anti-HLA antibodies, using the Luminex platform. Deceased donor kidneys were allocated based on HLA compatibility, with a single priority criterion related to the imminent technical impossibility to obtain access to perform any type of dialysis. We excluded recipients of simultaneous kidney and pancreas transplants, pediatric kidney transplant recipients, patients who received basiliximab induction and patients who did not receive induction or with no available data on induction and those who received CNI-free immunosuppressive regimens.

### Immunosuppression protocols

#### Induction therapy

During the first period, all patients with PRA > 50% or with other risk factors such as low HLA compatibility (more than 3 HLA mismatches), long cold ischemia time (higher than 24 hours), priority criterion (priority status attributed to a patient with imminent lack of access for peritoneal hemodialysis during the allocation process), and/or donor type (expanded donor criteria) received the standard of care induction therapy consisting of five 1 mg/kg doses of rATG. The initial 1.0 mg/kg dose of rATG, administered intravenously over 8 hours, beginning within the first 24 hours after graft revascularization. The subsequent 4 daily doses were adjusted based on the peripheral lymphocyte counts (< 100 cells/mm^3^: hold the dose; 100–150 cells/mm^3^: reduce 25 mg; 150–300 cells/mm^3^: maintain the dose; >300 cells/mm^3^: increase 25 mg). During the second period, all patients, regardless of the value of PRA, received a single 3mg/kg dose of rATG, administered intravenously over 10 hours, beginning within the first 24 hours after graft revascularization. In both periods, the rATG dose was based on current dry weight, rounded to the 25 mg vial size.

#### Maintenance therapy

Patients received tacrolimus (TAC) 0.1 mg/kg twice daily starting on day 1, with doses adjusted to maintain whole blood trough concentrations between 5–15 ng/ml, in combination with mycophenolate sodium 720 mg twice daily or azathioprine 2 mg/kg daily. Patients received TAC 0.05 mg/kg twice daily, with doses adjusted to maintain blood concentrations between 3–5 ng/ml, in combination with everolimus 1.5 mg twice daily, to maintain whole blood trough concentrations between 3 to 8 ng/ml. Patients received cyclosporine (CsA) 5 mg/kg twice daily, with doses adjusted to maintain whole blood trough concentrations between 100 to 300 ng/ml in combination with mycophenolate sodium 720 mg twice daily or azathioprine 2 mg/kg daily. All recipients were initiated on intravenous methylprednisolone (500 mg) intraoperative, followed by 0.5 mg/kg oral PRED, tapered to daily 5 mg doses by the end of the first month. Maintenance drug combination was selected based on perceived risk assessment, considering the previous transplant outcomes and the immunological risk of the retransplant.

### Prophylaxis

All patients received a 5-day course of 400 mg of albendazole and continuous use of sulfamethoxazole-trimethoprim. None of the patients received pharmacological prophylaxis for CMV infection. Preemptive strategy was used for donor (+) and recipient (-) CMV serostatus combination (D+/R-), for patients receiving mycophenolate sodium and after treatment of acute rejection AR episodes. The other patients were monitored at the physician discretion. The pre-emptive therapy consisted of every other week monitoring the viral replication, from the third week after transplantation, until the end of the third month, using the CMV pp65 antigenemia assay [13]. During the study period, the CMV antigenemia test was performed in a single laboratory using the CMV Brite Turbo kit, according to the manufacturer’s recommendations (IQ® Products, Groningen, Netherlands).

### Acute rejection

Treated acute rejection (tAR) included clinical acute rejections and biopsy proven acute rejection (BPAR). Clinical acute rejections were defined as graft dysfunction, without histological evidence of rejection and treated with methylprednisolone for at least three days. All episodes BPAR were adjudicated and retrospectively reclassified based on the Banff 2019 criteria. The cumulative survival-free of BPAR (≥IA) were compared over 3 years of follow up.

### Graft loss and mortality

The cumulative incidence of graft loss, death-censored graft loss and mortality were compared over 3 years of follow up. Graft loss was defined as the need for permanent return to dialysis. Loss to follow-up due to referral to another transplant center was defined by the lack of information for more than 6 months.

### Safety outcomes

We selected three safety surrogate outcomes to compare the differential effect of the two rATG doses, namely, complications associated with the surgical procedure, early hospital admission for any cause and CMV events.

### Surgical complications

We evaluated the incidence, timing and recurrence of surgical complications between the two groups.

### Early hospital readmission

The incidence and specific causes of EHR, defined as all readmissions within 30 days of initial hospital discharge, were compared between the two groups.

### CMV infection or disease

CMV infection was defined as the presence of more than 10 infected cells in a total of 200,000 peripheral blood neutrophils in asymptomatic patients, based on CMV pp65 antigenemia assay. CMV disease was diagnosed based on the presence of CMV-related signs or symptoms including fever, asthenia, myalgia, leukopenia, thrombocytopenia, or liver enzymes abnormalities, and the presence of any number of CMV pp65 infected cells. CMV infection or disease was treated with intravenous ganciclovir for at least 14 days with weekly monitoring of viral replication. Treatment was continued for 1 week, after the first negative CMV pp65 antigenemia test.

### DGF and renal function and histology

Delayed graft function was defined as the need of dialysis during the first week after transplantation, excluding a single dialysis for hypervolemia and/or hyperkalemia. Duration of DGF was measured from the time of transplant to the last dialysis. Renal function was assessed by the estimated glomerular filtration rate (eGFR, mL/min/1.73 m^2^) calculated with the Chronic Kidney Disease Epidemiology (CKD-EPI) study equation. Surveillance graft biopsies were performed during the DGF period and in patients with incomplete renal function recovery, as well as in all episodes of graft dysfunction.

### Primary endpoint

The primary endpoint was to compare the incidence of efficacy failure, defined by first biopsy proven acute rejection (≥ IA), graft loss, or death between the groups, at 12 months.

### Secondary endpoints

Secondary endpoints included the comparison of the cumulative incidence of first biopsy proven acute rejection (≥ IA), graft loss, death-censored graft loss and mortality over 36 months. We also analyzed the incidence and severity of treated acute rejection episodes, the incidence of surgical complications, early hospital readmission and CMV events during the first 12 months. Finally, we compared the incidence and duration of DGF, the trajectories of renal function, and proteinuria up to 36 months of follow up.

### Statistical analysis

Categorical variables were expressed as absolute frequency and percentages and the differences between groups were performed using the chi-square test or Fisher’s exact test. Continuous variables were presented as mean and standard deviation or medians (interquartile range [IQR]), depending on normality. Differences between groups were identified using the Mann-Whitney test or Student’s t-test. Renal function (eGFR) trajectories were compared up to 36 months using a generalized model for repeated measures, without and with imputation of a value of "zero" in cases of graft loss, and with the last observation carried forward analysis (LOCF) for patients who died or were loss to follow up. Multiple linear regression was used to identify independent risk factors associated with 1-month eGFR.

Considering the non-inferiority margin of 15% for the experimental rATG-3 group compared to the standard rATG-5 group for the primary endpoint of efficacy failure at 12 months, a significance level of 5%, a statistical power of 80%, and the efficacy failure of 25% in the control arm, 208 patients are required to be 80% sure that the upper limit of a one-sided 95% confidence interval. We used Cox proportional hazard model to compare hazard functions using treatment group as factor. Non-inferiority was determined if upper confidence limit of HR was less than the non-inferiority margin then non-inferiority using Wald’s confidence interval. The survival curves were obtained by the Kaplan-Meier method and the differences identified by the Log-Rank test, censored for patients with loss to follow-up. All comparisons were made using the intention to treat population, defined as patients receiving at least of dose of rATG in both groups.

All statistical analyzes were performed using the SPSS v.21 program (SPSS inc., Chicago, IL, USA), with two-tailed hypothesis testing and a α of 0.05 as a criterion for statistical significance.

## Results

### Study population

There were 4030 kidney transplants between January 1, 2010 and June 16, 2014, of which 203 (5%) were retransplants, while among 2098 procedures between June 17, 2014 and October 9, 2016, 139 (6.6%) were retransplants. We excluded 103 retransplants in the first period and 29 in the second period who did not receive induction therapy with rATG (S1 Fig). Therefore, we identified 100 consecutive retransplants who received 5 mg/kg and 110 receiving 3 mg/kg rATG. Patients were relatively young despite long time on dialysis and 13% had priority criterion. About 44% had received the first kidney transplant from a deceased donor and a similar proportion had undergone previous graft nephrectomy. The majority was sensitized and receiving a second transplant from a deceased donor. The proportion of patients with PRA class I or class II >50% was 65% (n = 65) in the rATG-5 group and 56% (n = 62) in the rATG-3 group. There were no differences in median cPRA (50% vs. 44%) but patients in the rATG-3 had lower median PRA class I. Importantly, 73% received a zero HLA DR mismatch and 14% a zero HLA A, B, DR mismatch kidney retransplants. Median KPDI was 60% and 19% were extended criteria donors. There was an imbalance in the distribution of donor/recipient mismatches favoring the rATG-5 group. The degree of acute kidney injury, as measured by the difference between the final and initial creatinine (Δ), was higher in the rATG-3 group. Finally, kidneys were transplanted with a median cold ischemia time between 22 and 24 hours (Table 1).

**Table 1:** Demographic characteristics of the study population.

Parameters	rATG-5 (n = 100)	rATG-3 (n = 110)	p value
**Recipient**			
Age (years), median (IQR)	40 (32.5; 47)	40 (30; 50)	0.873
Sex (male), n (%)	54 (54)	68 (61.8)	0.251
Donor/recipient			0.043
*Donor male/recipient male*	35 (35)	37 (33.5)	
*Donor female/recipient male*	19 (19)	31 (28)	
*Donor male/recipient female*	30 (30)	17 (15.5)	
*Donor female/recipient female*	16 (16)	25 (23)	
Race, n (%)			0.657
*White*	51 (51)	57 (51.8)	
*Black*	16 (16)	13 (11.8)	
*Other*	33 (33)	40 (36.4)	
CKD etiology, n (%)			0.541
*Hypertension*	11 (11)	10 (9.1)	
*Glomerulonephritis*	34 (34)	42 (38.2)	
*Diabetes Mellitus*	3 (3)	2 (1.8)	
*In determined*	36 (36)	42 (38.2)	
*Urologic*	6 (6)	9 (8.2)	
*Polycystic kidney disease*	1 (1)	2 (1.8)	
*Other*	9 (9)	3 (2.7)	
Renal replacement therapy, n (%)			0.491
*Hemodialysis*	83 (83)	83 (7.,5)	
*Peritoneal dialysis*	2 (2)	3 (2.7)	
*Hemodialysis + Peritoneal dialysis*	15 (15)	23 (20.9)	
*Conservative*	0	1 (0.9)	
Viral serology, n (%)			0.278
HIV	1 (1)	1 (0.9)	
HBV	0	4 (3.6)	
HCV	9 (9)	8 (7.3)	
CMV (donor/recipient)			0.213
*Donor (+)/recipient (-)*	2 (2)	0 (0)	
*Donor unknown/recipient (-)*	1 (1)	1 (0.9)	
*Donor (+/-)/recipient (+)*	95 (95)	109 (99.1)	
*Donor unknown/recipient unknown*	2 (2)	0 (0)	
Time on dialysis (months), median (IQR)	71 (28; 118)	53 (26; 121)	0.238
Priority criterion, n (%)	16 (16)	12 (10.9)	0.278
Previous transplant (deceased), n (%)	44.9 (44)	44 (40)	0.475
Previous graft nephrectomy	46 (46)	50 (45.5)	0.937
Current kidney transplant, n (%)			0.356
*Second*	95 (95)	101 (91.8)	
*Third*	5 (5)	9 (8.2)	
cPRA Class I, median (IQR)	58.5 (19; 98)	31.5 (0; 76)	0.045
cPRA Class II, median (IQR)	23 (0; 57.5)	18 (0; 53)	0.509
cPRA, median (IQR)	50 (21.75; 78.25)	44 (4.4; 83.6)	0.111
HLA mm, median (IQR)	2 (1;3)	2 (1;3)	0.803
zero HLA A mm, n (%)	29 (29)	29 (26.4)	0.392
zero HLA B mm, n (%)	28 (28)	29 (26.4)	0.455
zero HLA DR mm, n (%)	75 (75)	78 (70.9)	0.305
zero HLA A, B, DR, n (%)	15 (15)	14 (12.7)	0.390
**Donor**			
Age (years), median (IQR)	42 (31; 53)	45.5 (37; 54)	0.421
Sex (male), n (%)	65 (65)	54 (49.1)	0.020
Race, n (%)			0.826
*White*	60 (60)	65 (59.1)	
*Black*	14 (14)	13 (11.8)	
*Other*	26 (26)	32 (29.1)	
Deceased donor, n (%)	94 (94)	99 (90)	0.289
*ECD criteria*, *n (%)*	22 (22)	17 (15.4)	0.281
Cause of death, n (%)			0.767
*Cerebrovascular*	56 (59.6)	60 (60,6)	
*Trauma*	31 (33)	31 (31,3)	
*Tumor SNC*	2 (2.1)	1 (1)	
*Anoxia*	3 (3.2)	2 (2)	
*Other*	2 (2.1)	5 (5.1)	
Previous cardiac arrest, n (%)	18 (19.1)	14 (14.1)	0.350
Use of vasoactive drug, n (%)	82 (87.2)	88 (88,9)	0.723
Infection, n (%)	37 (39.4)	46 (46.5)	0.319
Diabetes, n (%)	5 (5.3)	4 (4)	0.674
Hypertension, n (%)	31 (33)	38 (38.4)	0.434
Initial creatinine, mg/dl, median (IQR)	1.0 (0.8; 1,2)	0.8 (0.59; 1,01)	0.070
Final creatinine, mg/dl, median (IQR)	1.28 (0.74; 1,82)	1.4 (0.6; 2,2)	0.134
Δ creatinine, mg/dl, median (IQR)	0.2 (-0.10; 0.85)	0.5 (0; 1.4)	0.019
KDPI %, median (IQR)	60 (36; 84)	63 (46; 80)	0.245
Cold ischemia time (hours) median (IQR)	22 (19; 25)	24 (19,5; 28,5)	0.060

Initial creatinine value is the first value obtained at the time of hospital admission of the potential donor. Final creatinine is the last creatinine value before organ recovery. Delta creatinine is an arbitrary measure of acute kidney injury. Δ creatinine = final creatinine–initial creatinine (75 paired samples in each group).

r-ATG: rabbit antithymocyte globulin; IQR: interquartile interval; CKD: Chronic Kidney Disease; HIV: human immunodeffiency virus; HBV: hepatitis B virus; HCV: hepatitis C virus; CMV: cytomegalovirus; cPRA: calculated panel reactive antibody; HLA mm: human leukocyte antigen mismatches; ECD: Expanded criteria donor; CNS: central nervous system; KDPI: Kidney Donor Profile Index.

### Immunosuppression

As per protocol, there was a significant difference in the median dose of rATG (5.3 vs. 3.01 mg/kg, p<0.0001). In the rATG-5 group only 4 patients received less than 3 mg/kg, 32 received between 3 and 5 mg/kg, 32 received between 5 and 6 mg/kg, and 32 more than 6 mg/kg (S1 Table). The majority of the patients received tacrolimus in combination with mycophenolate sodium in both groups. Mean tacrolimus whole blood trough concentrations were lower in the rATG-5 at 1 month (10.2±3.6 vs. 11.6±4.5 ng/ml, p = 0.018) and 12 months (7.8±2.8 vs. 9.1±3.5 ng/ml, p = 0.008) while mean prednisone doses were higher at 1 month (17.4±6.4 vs. 12.4±5.4 mg/day, p<0.0001), 3 months (8.2±5.8 vs. 5.2±1.4 mg/day, p<0.0001) and 6 months (6.1±3.6 vs. 5.2±0.9 mg/day, p = 0.039) compared to rATG-3. There were no differences in mean mycophenolate sodium doses during the first 12 months after transplantation (Table 2).

**Table 2:** Immunosuppression.

Parameters	rATG-5 (n = 100)	rATG-3 (n = 110)	p value
rATG dose (mg/kg), median (IQR)	5.30 (4.44; 6.16)	3.01 (2.9; 3.11)	0.000
Immunosuppression, n (%)			
*Tacrolimus*	99 (99)	109 (99)	0.946
*Cyclosporine*	1 (1)	0	0.293
*Mycophenolate*	98 (98)	98 (89)	0.010
*Azathioprine*	0	3 (2)	0.096
*Everolimus*	2 (2)	9 (8.2)	0.045
Tacrolimus (ng/ml), mean±SD			
*Month 1*	10.2±3.6	11.6±4.5	0.018
*Month 3*	8.6±3.0	9.7±4.9	0.117
*Month 6*	8.2±2.8	9.0±3.7	0.124
*Month 12*	7.8±2.8	9.1±3.5	0.008
Mycophenolate (mg/day), mean±SD			
*Month 1*	1349±230	1358±220	0.785
*Month 3*	1203±335	1231±325	0.604
*Month 6*	1228±318	1160±3511	0.306
*Month 12*	1221±313	1117±350	0.110
Prednisone (mg/day), mean±SD			
*Month 1*	17.4±6.4	12.4±5.4	0.000
*Month 3*	8.2±5.8	5.2±1.4	0.000
*Month 6*	6.1±3.6	5.2±0.9	0.039
*Month 12*	5.6±2.6	5.3±2.0	0.187

r-ATG: rabbit antithymocyte globulin; IQR: interquartile range; SD: standard deviation.

### Efficacy failure

**Fig 1:**
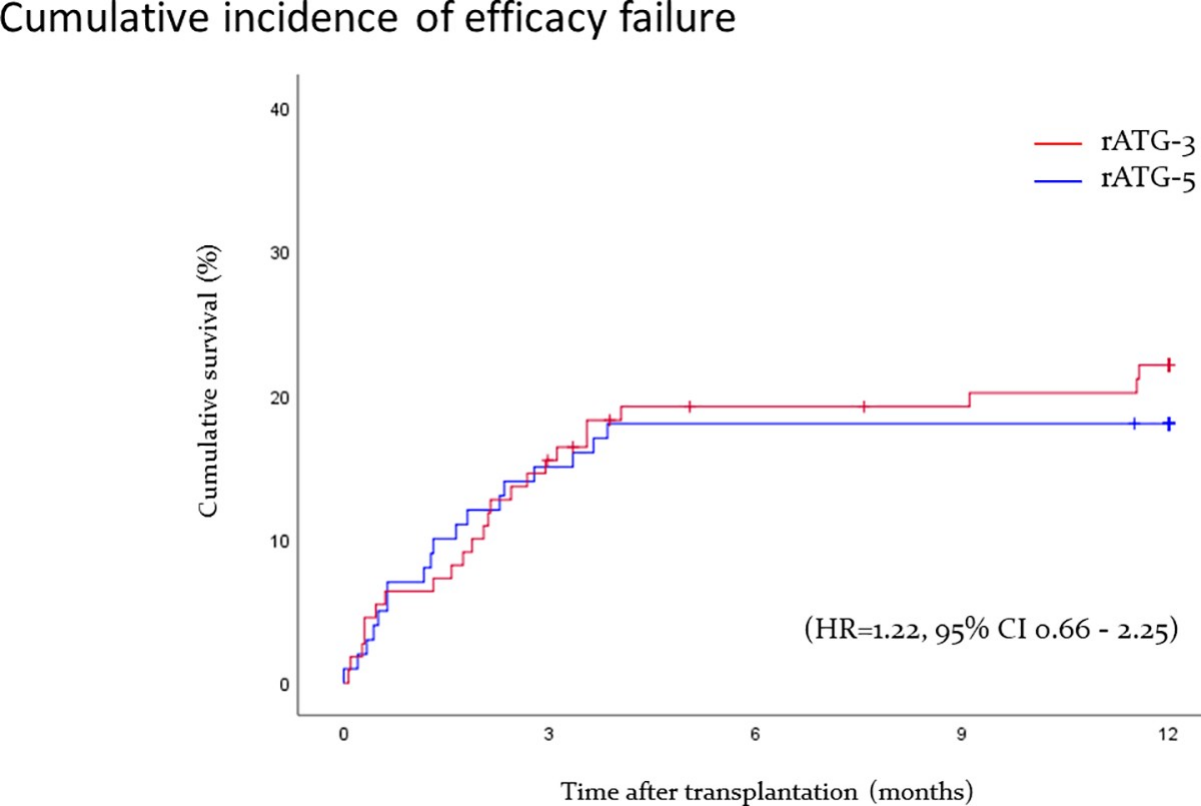
Cumulative survival-free of efficacy failure (first biopsy proven acute rejection, graft loss, death), in patients undergoing retransplantation receiving 5 or 3 mg/kg rATG induction therapy.

There was no significant difference in the incidence of efficacy failure at 12 months (18.0% in the rATG-5 vs. 21.8% in the rATG-3 group, HR = 1.22, 95% CI 0.66–2.25, Table 3, Fig 1). There was no difference in the incidence, severity, timing, and type of treatment or recurrent episodes of acute rejection. Overall, there was no difference in the total number of treated acute rejection episodes (rATG-5, n = 30 vs. rATG-3, n = 28), yet the total number of antibody mediated and mixed acute rejection episodes in the rATG-3 was higher compared to rATG-5 group (11 vs. 4, Table 3), respectively. There was also no difference in the incidence or specific causes of graft loss and death.

**Table 3:** Efficacy parameters at 12 months.

Parameters	rATG-5 (n = 100)	rATG-3 (n = 110)	p value
Efficacy failure, n (%)*	18 (18)	24 (21.8)	0.522
First BPAR ≥ IA, n (%)	10 (10)	15 (13.6)	0.55
First BPAR, n (%)	20 (20)	19 (17.3)	0.616
Severity *n (%)*			0.305
*Borderline*	10 (43.5)	4 (19.0)	
*IA*	4 (17.4)	6 (28.6)	
*IB*	3 (13.0)	1 (4.8)	
*IIA*	2 (8.7)	2 (9.5)	
*ABMR*	1 (4.3)	3 (14.3)	
*Mixed*	0 (0)	3 (14.3)	
Clinical acute rejection, n	3 (13)	1 (5.0)	
First tAR, n (%)	23 (23)	20 (18.2)	0.485
Time to first tRA, days, median (IQR)	35 (13; 83)	39 (10; 106)	1.000
Treatment, n (%)			0.449
*Methylprednisolone*	20 (87.0)	13 (61.9)	
*rATG/methylprednisolone*	2 (8.6)	4 (19.2)	
*Plasmapheresis/Immunoglobulin*	1 (4.3)	3 (14.3)	
*Graft removal*	0	1 (4.8)	
Patients with recurrent AR, n (%)	5 (21.7)	6 (28.6)	0.601
Second rejection, n (%)			0.239
*Borderline*	1 (20.0)	2 (33.3)	
*IA*	0 (0)	0 (0)	
*IB*	1 (20.o)	0 (0)	
*IIA*	0 (0)	0 (0)	
*ABMR*	2 (40.0)	0 (0)	
*Mixed*	1 (20.0)	4 (66.7)	
Third rejection, n (%)			0.466
*Borderline*	1 (25)	0 (0)	
*IA*	1 (25)	0 (0)	
*IB*	0 (0)	1 (50.0)	
*IIA*	0 (0)	0 (0)	
*ABMR*	0 (0)	0 (0)	
*Mixed*	0 (0)	1 (50.o)	
Clinical acute rejection	2 (50.0)	0 (0)	
Graft loss, n (%)	8 (8)	10 (9)	0.873
*FSFS recurrence*	1 (8.3)	1 (7.1)	
*Vascular thrombosis*	3 (37.5)	2 (14.3)	
*Primary Nonfunction*	1 (8.3)	1 (7.1)	
*Refractory acute rejection*	1 (8.3)	2 (14.3)	
*Pyelonephritis*	0	2 (14.3)	
*Sepsis*	1 (8.3)	1 (7.1)	
*Withdrawal of immunosuppression*	1 (8.3)	1 (7.1)
Death, n (%)	5 (5)	4 (3.6)	0.626
*Sepsis*	4 (80)	2 (50)	
*Acute myocardial infarction*	1 (20)	1 (25)	
*Traffic accident*	0	1 (25)	
Loss to follow up, n (%)	1 (1)	5 (4.5)	0.117

* HR = 1.22, 95% CI 0.66–2.25.

r-ATG: rabbit antithymocyte globulin; BPAR: biopsy proven acute rejection; ABMR: antibody mediated rejection; IQR: interquartile range; tAR: treated acute rejection; AR: acute rejection; FSGS: focal and segmentar glomerulosclerosis; HR: hazard ratio; CI: confidence interval.

Because 36 patients in the rATG group did not receive the intended total dose due to adverse events, a subgroup analysis revealed a higher incidence of first treated acute rejection (30.6% vs. 18.8%, p = 0.178) and first BCAR ≥ IA (19.4 vs. 4.7%, p = 0.033) comparing patients receiving < 5 mg/kg (n = 36) or ≥ 5 mg/kg (n = 64) total dose of rATG in the rATG-5 group.

**Fig 2:**
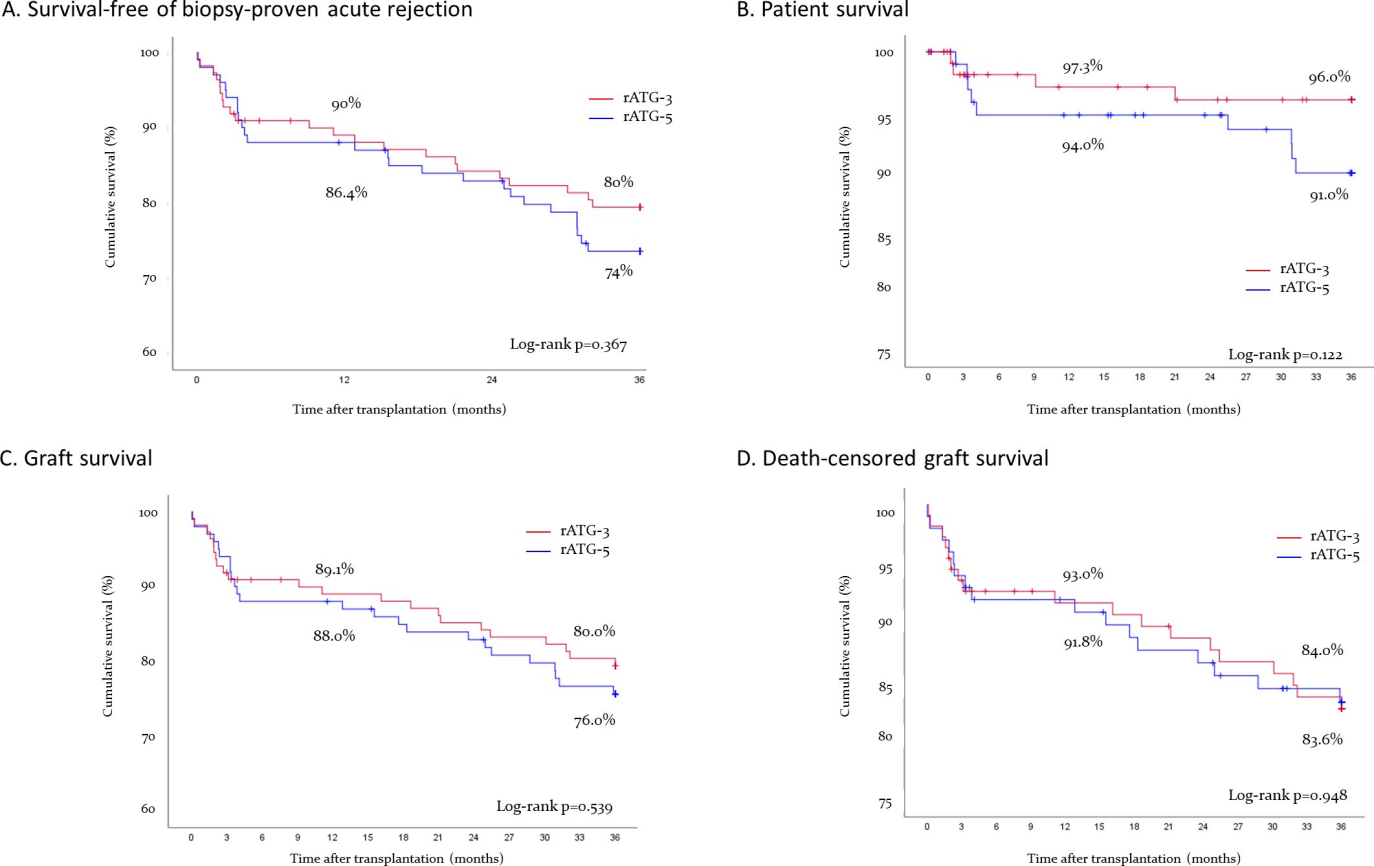
Cumulative survival-free of first biopsy proven acute rejection (A), patient survival (B), graft survival (C) and death-censored graft survival (D), in patients undergoing retransplantation receiving 5 or 3 mg/kg rATG induction therapy.

The number of treated acute rejection episodes and graft losses from month 12 to month 36 were comparable but a higher number of deaths were observed in the rATG-5 compared to rATG-3 (S2 Table). There were no differences in cumulative survival-free of first BPAR (12 months: 90.0% vs. 86.4%, p = 0.477; 36 months: 74 vs. 80%, p = o.367, Fig 2A), patient survival (12 months: 94.0% vs. 97.3%, p = 0.419; 36 months: 91.0% vs. 96.4%, p = 0.122, Fig 2B), graft survival (12 months: 88.0 vs. 89.1%, p = 0.843; 36 months: 76.0 vs. 80.0%, p = 0.539, Fig 2C), and death-censored graft survival (12 months: 93.0% vs. 91.8%, p = 0.944; 36 months: 84.0% vs. 83.6%, p = 0.948, Fig 2D) between the rATG-5 and rATG-3 groups, respectively.

### Safety assessments

Patients in the rATG-3 group tended to show a lower incidence of surgical complications compared to rATG-5, although there were no differences in the length of hospital stay for the transplant surgery or in the incidence and specific causes of early hospital readmission. The prevalence of high risk donor positive recipient negative CMV serostatus (D+/R-) combination was low and comparable between the two groups (Table 1). Using only preemptive therapy, there were no differences in the incidence of CMV events (48% vs. 40%, p = 0.19). None of the 11 patients receiving everolimus in both groups developed CMV infection, as well as the 3 patients who received azathioprine in the rATG-3 group. There was no difference in the incidence of CMV infection comparing patients receiving CNI/MPA in the rATG-5 (49%) and in the rATG-3 (45%). Importantly, most events were CMV infection and there were no episodes of CMV tissue invasive disease (Table 4).

**Table 4:** Surgical complications, early hospital readmission and CMV events.

Parameters	rATG-5 (n = 100)	rATG-3 (n = 110)	p value
Patients with surgical complication, n (%)	25 (25)	20 (18.2)	0.229
Time to first surgical complication (days), median (IQR)	20 (5.5; 34.5)	12.5 (0; 26.5)	0.264
Recurrent surgical complication, n (%)	5 (20)	4 (20)	1.000
Length of hospital stay for the transplant surgery, median (IQR)	12.5 (4; 21)	11 (6; 16)	0.134
Early hospital readmission, n (%)	27 (27.8)	31 (29.5)	0.457
Etiology, n (%)			0.699
*Infection*	34 (61.8)	39 (63.9)	
*Acute rejection*	2 (3.6)	5 (8.2)	
*Surgical complication*	9 (16.4)	8 (13.1)	
*Cardiovascular*	6 (10.9)	5 (8.2)	
*Neoplasia*	1 (1.8)	0	
*Toxicities*	3 (5.5)	2 (3.3)	
*Other*	0	2 (3,3)	
First CMV event, n (%)	48 (48)	44 (40)	0.190
*Infection*	38 (78)	33 (75)	
*Disease*	11 (22)	11 (25)	
Time to first CMV event, days, median (IQR)	43 (30.7; 55.2)	43,5 (26; 61)	0,408
Recurrent CMV event, n (%)	9 (18.4)	12 (27.3)	0,308

r-ATG: rabbit antithymocyte globulin; IQR: interquartile range; CMV: cytomegalovirus.

### Delayed graft function, renal function and histology

**Fig 3:**
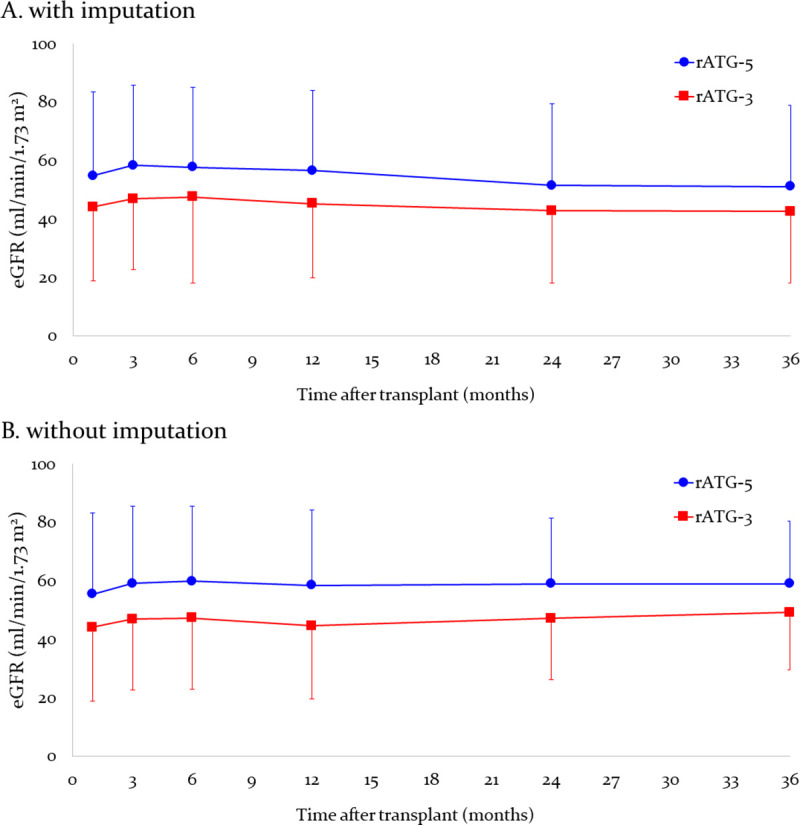
Renal function trajectories (eGFR) over the first three years after kidney retransplantation, comparing patients receiving 5 and 3 mg/kg rATG induction therapy. Data represents mean and standard deviation of eGFR with imputation (zero value for patients with graft loss, and last observation carried forward for patients who died or were loss to follow up).

There were no differences in the incidence and duration of DGF between the two groups. Patients in the rATG-5 group showed a significantly higher mean eGFR from month 1 to month 36 compared to patients in the rATG-3 group, with and without imputation, and mean urinary protein concentration was higher in the rATG-3 group at months 1, 24 and 36 (Table 5). The difference in eGFR was observed in patients without acute rejection, from month 1 (56.96±29.85 vs. 44.96±25.60 ml/min/1.73 m^2^, P<0.001) to month 36 (53.25±28.22 vs. 44.58±24.00, p<0.001, respectively (S3 Table). There were no significant differences in mean eGFR of recipients of living donor allografts, and of deceased donors allografts with KDPI < 20% (Table 5, S2 Fig). Finally, there were no significant differences in renal function trajectories over the study period (Fig 3). The differences in eGFR at month 1 and month 12 were influenced by the unbalanced distribution of donor and recipient sex mismatches (S4 Table). Yet only the rATG-3 group (OR 2.93) and DGF (OR 2.46) were associated with inferior (< 48 ml/min/1.73 m^2^) eGFR at 1 month (Table 6).

**Table 5:** Incidence and duration of DGF, trajectories of renal function and proteinuria.

Parameters	rATG-5 (n = 100)	rATG-3 (n = 110)	p value
DGF, n (%)	59 (59.6)	64 (58.7)	0.897
*Duration of DGF*, *days*, *median (IQR)*	4 (0; 10)	2 (0; 8)	0.188
eGFR (ml/min/1.73 m^2^, with imputation)			
*Month 1*	54.7±28.8	44.1±25.3	p = 0.005
*Month 3*	58.3±27.5	46.9±24.4	p = 0.001
*Month 6*	57.7±27.4	47.5±29.4	p = 0.05
*Month 12*	56.5±27.5	45.2±25.5	p = 0.002
*Month 24*	51.4±27.9	42.8±24.7	p = 0.019
*Month 36*	51.1±27.7	42.5±24.5	p = 0.019
eGFR (ml/min/1.73 m^2^, without imputation)			
*Month 1*			
*All*	55.4±27.9	44.1±25.3	p = 0.003
*Living donors*	69.4±19.3	72.5±23.5	p = 0.799
*Deceased donors without DGF*	60.0±27.16	49.3±26.2	p = 0.157
*Deceased donors KDPI 0–20%*	54.8±31.1	58.6±26.4	p = 0.548
*Month 3*	59.1±26.4	46.9±24.4	p = 0.001
*Month 6*	59.9±25.6	47.3±24.4	p = 0.001
*Month 12*			
*All*	58.5±25.8	44.6±25.0	p = 0.000
*Living donors*	73.8±16.4	71.0±16.8	p = 0.761
*Deceased donors without DGF*	58.5±24.6	44.4±24.3	p = 0.022
*Deceased donors KDPI 0–20%*	66.1±27.9	52.4±19.6	p = 0.127
*Month 24*	59.0±22.5	47.1±20.9	p = 0.000
*Month 36*	58.9±21.4	49.1±19.5	p = 0.003
Proteinuria (g/mL)			
*Month 1*	0.42±0.76	0.63±0.85	p = 0.001
*Month 3*	0.40±0.76	0.40±0.51	p = 0.087
*Month 6*	0.42±1.07	0.28±0.39	p = 0.639
*Month 12*	0.50±1.72	0.40±0.83	p = 0.306
*Month 24*	0.26±0.52	0.51±0.99	p = 0.003
*Month 36*	0.38±0.81	0.70±2.40	p = 0.008

DGF: delayed graft function; IQR: interquartile range; eGFR: estimate graft filtration rate; KDPI: Kidney Donor Profile Index.

**Table 6:** Multivariable logistic regression analysis for 1 month eGFR < 48 ml/min/1.73 m^2^ (n = 210).

Parameters	HR	95% C.I.		Sig.
		Inferior	Superior	
KDPI, %	1.012	0.998	1.026	0.083
CIT, hours	0.975	0.924	1.029	0.352
SEX (M-M, reference)				0.611
F-F	1.051	0.429	2.576	0.913
M-F	1.102	0.464	2.619	0.263
F-M	0.263	0.614	0.262	0.826
Time on dialysis, months	1.005	0.999	1.011	0.132
cPRA, %	0.987	0.412	2.362	0.976
Induction era (rATG-5, reference)	3.471	1.829	6.589	0.000
DGF (no, reference)	2.549	1.300	4.997	0.006

eGFR: estimate graft filtration rate; HR: hazard ratio; CI: confidence interval; KDPI: Kidney Donor Profile Index; CIT: Cold ischemia time; M: Male; F: Female; cPRA: calculated panel reactive antibody; rATG: rabbit antithymocyte globulin; DGF: delayed graft function.

A higher proportion of patients in the rATG-3 group required at least one biopsy, either early or late after transplantation, and the number of biopsies per patient was also higher compared to patients in the rATG-5 group. Biopsies of patients in the rATG-3 group showed higher cg scores, early (0–3 months) and late (4–36 months) after transplantation. Nonetheless, there was no statistical difference in mean total Banff scores, either in biopsies performed within the first 3 months or afterwards (Table 7).

**Table 7:** Summary of kidney transplant pathology of all biopsies up to 36 months.

Parameters	rATG-5 (n = 100)	rATG-3 (n = 110)	p value
Patients with biopsy, n (%)	47 (47)	78 (70.9)	0.001
Number of biopsies per patient, median (IQR)	1 (1; 2)	1 (1; 3)	0.007
Number of biopsies (0–3 months)	45	110	
Chronic Banff scores (0–3 months), mean±SD			
cg	0.0 ± 0.0	0.1 ± 0.10	0.016
*ct*	0.21 ± 0.41	0.42 ± 0.65	0.112
*ci*	0.21 ± 0.41	0.46 ± 0.65	0.112
*cv*	0.39 ± 0.55	0.48 ± 0.72	0.131
*ah*	0.50 ± 0.65	0.41 ± 0.68	0.128
*cg+ct+ci+cv*	0.81 ± 1.10	1.33 ± 1.68	0.229
Number of biopsies (4–36 months)	41	77	
Chronic Banff scores (4–36 months), mean±SD			
cg	0.0 ± 0.0	0.04 ± 0.26	0.044
*ct*	1.17 ± 0.79	1.31. ±0.87	0.173
*ci*	1.17 ± 0.79	1.31. ± 0.87	0.173
*cv*	0.97 ± 0.97	0.97 ± 0.93	0.199
*ah*	0.79 ± 0.88	0.76 ± 0.84	0.176
*cg+ct+ci+cv*	3.22 ±2.15	3.61 ± 2.29	0.477

r-ATG: rabbit antithymocyte globulin; IQR: interquartile range; SD: standard deviation; cg: glomerular basement membrane double contours; ct: tubular atrophy; ci: interstitial fibrosis; cv: vascular fibrous intimal thickening; ah: arteriolar hyalinosis.

## Discussion

This sequential cohort experiment showed that the use of a single 3 mg/kg dose of rATG was associated with similar efficacy for the prevention of acute rejection, graft loss and death compared to the traditional 5 days course of 1 mg/kg rATG, over the first 3 years in recipients of repeat kidney transplants without preformed HLA A, B, and DR DSA. The observed 3-years graft, death-censored graft and patient survivals were comparable with that reported by the SRTR data [14]. These data are concordant with two other retrospective analysis in low-risk [15] and high-risk [16] kidney transplant recipients in our institution.

The demographic characteristics of the study population were comparable regarding key immunological risk factors. Furthermore, given the allocation policy and the large size of the local waiting list, 14% received a zero HLA A, B, DR mismatched and 73% zero HLA DR mismatched allografts, suggesting a further long-term benefit for this population [17, 18]. Recent studies have shown that the risk of antibody-mediated rejection and premature graft loss is associated with preformed DSA and not with the level of sensitization, measure by cPRA, as previously suggested [19]. Also, recent data suggest that, in the absence of detectable preformed DSA, reexposure to mismatched HLA antigens present in the first kidney transplant is not associated with de novo DSA development, rejection, or allograft loss [5]. Finally, the proportion of patients with previous allograft nephrectomy, which has been associated with an increased risk of development of anti-HLA antibodies [20], was comparable between the two groups.

In this cohort of patients we did not compare the pharmacodynamic effect of the two dosing rATG regimens using serial lymphocyte counts in the peripheral blood during the first year. A previous study showed comparable T, B and NK cell depletion up to one month in a cohort of patients receiving 3 or 6 mg/kg of rATG that persisted up to 12 months only in the 6 mg/kg dose group [21]. Importantly, only 64% of the patients in the rATG-5 receive the full 5 mg/kg course of rATG. This observation is frequent as shown in the seminal study by Brennan D et al., where only 68.6% of the patients received the intended five doses of rATG [22]. We also observed a similar pattern analyzing this strategy in a larger cohort of high-risk kidney transplant recipients [16]. The reasons to reduce the intended total dose are primarily safety issues, such as leukopenia, thrombocytopenia and surgical complications [22]. The trends towards higher incidence of acute rejection among patients receiving < 5 mg/kg in the rATG-5 group suggests that the inability to complete the intended 5 dose course, as a consequence of impeding toxicity, may increase the risk of acute rejection.

The incidence of first biopsy proven acute rejection was comparable to that observed in other cohorts [8]. This result is consistent with our previous study in high risk kidney transplant recipients, where a single 3 mg/kg rATG dose was associated with a decreased risk of CMV infection, without increasing the risk of acute rejection or compromising graft or patient survival compared to the traditional 6 mg/kg dose [16]. Yet, there were a higher number of antibody-mediated and mixed rejections in the rATG-3 group that might be associated, at least in part, with the presence of preformed anti-HLA C, DQ and DP antibodies [23].

Interestingly, the 2 mg/kg reduction in rATG dose was not associated with detectable safety benefits, including the number of surgical complications [24], early hospital readmissions and CMV infection. Nevertheless, a significant reduction in the incidence of CMV infection was observed in another high risk population using the same strategy in our center [16]. The overall incidence of CMV events was relatively high in both groups, but this is expected for patients receiving rATG without pharmacological prophylaxis [6]. Yet, despite the unique preemptive therapy strategy, more that 75% of the patients were treated for CMV infection and there were no cases of confirmed CMV invasive disease.

The relative high but comparable incidence of DGF [25] is relevant in the context of our current allocation policy, because the concomitant presence of preformed DSA has been associated with higher incidence of subclinical mixed and antibody-mediated rejection phenotypes and inferior 5-years graft survival [26]. An intriguing observation was noticed in the rate of renal function recovery after retransplantation. There was an early, evident and persistent 10 ml/min lower eGFR in the rATG-3 group. There are several evidences suggesting that the unbalanced rejection rates between the two groups did not influence kidney function. First, the incidence of treated rejection was low in both groups (20% rATG-5 vs. 17.3% rATG-3). Second, the difference in kidney function was noticed as early as 1 month, when not all rejections had occurred, and persisted throughout the follow up. Thirdly, the trajectories of kidney function in patients without treated acute rejection showed the same pattern as the overall population. Subtle differences in demographic characteristics may be involved, independently of the development of delayed graft function and acute rejection [27], including the effect of donor-recipient sex mismatches on renal function and graft survival [28, 29]. It is also conceivable that the higher rATG dose showed higher efficacy to reduce the ischemia/reperfusion injury, promoting more complete recovery of graft function, primarily among those patients receiving high KDPI kidneys with long cold ischemia time [30]. Another possibility would be a higher incidence of subclinical rejections. Yet, as a consequence of incomplete recovery of kidney function, a higher number of early biopsies were performed in patients in the rATG-3, as demonstrated by the difference in GFR at month 1.

Because of the perceived higher risk for acute rejection among recipients of retransplants, small increases in creatinine triggered the indication of a biopsy to rule out rejection. Despite undergoing a higher number of kidney biopsies, there was no statistical difference in the number of treated acute rejections or in the acute Banff scores. Finally, the analysis of the chronic Banff scores suggests that the difference in kidney function is associated, at least in part, by the chronic cg scores observed in both early and late biopsies. Considering the stable eGFR trajectories over the 36 months, the early and persistent low-grade proteinuria, and the higher cg Banff scores in the rATG-3 group, we speculate that the initial difference in eGFR was derived from the interaction between subtle unfavorable donor characteristics—including age, lower proportion of male and consequent unbalanced donor/recipient match, donor acute kidney injury as measured by Δ creatinine—and longer cold ischemia time, and recovery from ischemia reperfusion injury.

There are numerous limitations in our study. There are no data regarding the outcomes of previous first and second transplants, including the dose of rATG, the incidence of DGF, level of renal function, survival [2], and reexposure to HLA mismatched antigens [5], all associated with clinical outcomes after retransplantation [2]. We also do not have data on HLA C, DP and DQ mismatches nor the presence of DSA against these antigens, which are becoming more relevant for both short and long-term graft survivals [31]. Further, we did not obtained serial measurements peripheral blood lymphocytes and DSA to ascertain whether the development of de novo DSA is influenced by the pharmacodynamics effect of the rATG dose [32, 33]. Currently, we are not only obtaining these data, but also performing a flow cytometry crossmatch in all sensitized patients, during the allocation process. We also do not have serial data on poliomavirus viremia, although poliomavirus nephropathy was captured by the for cause biopsies. While we only performed biopsies to investigate graft dysfunction, protocol biopsies would improve the ability to detecting subclinical inflammation [26].

In summary, this retrospective sequential cohort study was unable to detect differences in the incidence of efficacy failure and short-term surrogate safety assessments by reducing the dose of rATG from 5 to 3 mg/kg, in recipients of repeat kidney transplants without preformed HLA A, B, DR DSA. Considering the time course of the early and persistent difference in eGFR and the biopsy findings, the lower renal function in the rATG-3 group appears to derive from unfavorable donor characteristics during recovery from ischemia reperfusion injury rather than lack of efficacy for the prevention of clinical and subclinical rejection.

## Supporting information

S1 Fig Patient disposition. (TIF)

S2 Fig Mean month 1 eGFR stratified by KDPI scores, according to the rATG dose. (TIF)

S1 Table Distribution of rATG doses in the rATG-5 group. (DOCX)

S2 Table Acute rejection, graft loss and deaths from 12 to 36 months. (DOCX)

S3 Table Trajectories of kidney function stratified by the presence of treated acute rejection (tAR). (DOCX)

S4 Table Renal function stratified by donor and recipient gender combinations. (DOCX)

S1 Appendix Informed consent form for solid organs and tissues donation–deceased donor–over 18 years-old. (DOCX)

S2 Appendix Term of authorization for kidney donation from transplantation. (DOCX)

S3 Appendix Master file. (XLSX)

## Abbreviations

BPAR: Biopsy proven acute rejection
CI: Confidence Interval
CKD-EPI: Chronic Kidney Disease Epidemiology Collaboration Equation
CMV: Cytomegalovirus
CsA: Cyclosporine
DGF: Delayed graft function
DSA: Donor specific antibody
eGFR: Estimated glomerular filtration rate
MFI: Mean fluorescence intensity
HBsAg: Hepatitis B Surface Antigen
HCV: Hepatitis C Virus
HIV: Human Immunodeficiency Virus
HLA: Human Leukocyte Antigen
OPTN/SRTR: Organ Procurement and Transplant Network/Scientific Registry of Transplant Recipients
PRA: Panel Reactive Antibody
rATG: rabbit antithymocyte globulin
USRDS: United States Renal Data System

## References

[1] Schold JD, Augustine JJ, Huml AM, O’Toole J, Sedor JR, Poggio ED. Modest rates and wide variation in timely access to repeat kidney transplantation in the United States. Am J Transplant. 2020;20(3):769–78. 10.1111/ajt.15646 31599065 PMC7204603

[2] Heaphy EL, Poggio ED, Flechner SM, Goldfarb DA, Askar M, Fatica R, et al. Risk factors for retransplant kidney recipients: relisting and outcomes from patients’ primary transplant. Am J Transplant. 2014;14(6):1356–67. 10.1111/ajt.12690 24731101

[3] Hart A, Lentine KL, Smith JM, Miller JM, Skeans MA, Prentice M, et al. OPTN/SRTR 2019 Annual Data Report: Kidney. Am J Transplant. 2021;21 Suppl 2:21–137. 10.1111/ajt.16502 33595191

[4] Clark S, Kadatz M, Gill J, Gill JS. Access to Kidney Transplantation after a Failed First Kidney Transplant and Associations with Patient and Allograft Survival: An Analysis of National Data to Inform Allocation Policy. Clin J Am Soc Nephrol. 2019;14(8):1228–37. 10.2215/CJN.01530219 31337621 PMC6682813

[5] Lucisano G, Thiruvengadam S, Hassan S, Gueret-Wardle A, Brookes P, Santos-Nunez E, et al. Donor-specific antibodies detected by single antigen beads alone can help risk stratify patients undergoing retransplantation across a repeat HLA mismatch. Am J Transplant. 2020;20(2):441–50. 10.1111/ajt.15595 31529621

[6] Hill P, Cross NB, Barnett AN, Palmer SC, Webster AC. Polyclonal and monoclonal antibodies for induction therapy in kidney transplant recipients. Cochrane Database Syst Rev. 2017;1(1):Cd004759. 10.1002/14651858.CD004759.pub2 28073178 PMC6464766

[7] Alloway RR, Woodle ES, Abramowicz D, Segev DL, Castan R, Ilsley JN, et al. Rabbit anti-thymocyte globulin for the prevention of acute rejection in kidney transplantation. Am J Transplant. 2019;19(8):2252–61. 10.1111/ajt.15342 30838775 PMC6767488

[8] Schold J, Poggio E, Goldfarb D, Kayler L, Flechner S. Clinical outcomes associated with induction regimens among retransplant kidney recipients in the United States. Transplantation. 2015;99(6):1165–71. 10.1097/TP.0000000000000507 25606788

[9] Singh N, Rossi AP, Savic M, Rubocki RJ, Parker MG, Vella JP. Tailored Rabbit Antithymocyte Globulin Induction Dosing for Kidney Transplantation. Transplant Direct. 2018;4(2):e343. 10.1097/TXD.0000000000000765 29464204 PMC5811272

[10] Tambur AR, Campbell P, Chong AS, Feng S, Ford ML, Gebel H, et al. Sensitization in transplantation: Assessment of risk (STAR) 2019 Working Group Meeting Report. Am J Transplant. 2020. 10.1111/ajt.15937 PMC7586936 32342639

[11] Zhang R. Donor-Specific Antibodies in Kidney Transplant Recipients. Clin J Am Soc Nephrol. 2018;13(1):182–92. 10.2215/CJN.00700117 28446536 PMC5753302

[12] Tedesco-Silva H, Felipe C, Ferreira A, Cristelli M, Oliveira N, Sandes-Freitas T, et al. Reduced Incidence of Cytomegalovirus Infection in Kidney Transplant Recipients Receiving Everolimus and Reduced Tacrolimus Doses. Am J Transplant. 2015;15(10):2655–64. 10.1111/ajt.13327 25988935

[13] Henrique Pinto C, Tedesco-Silva H Jr., Rosso Felipe C, Nicolau Ferreira A, Cristelli M, Almeida Viana L, et al. Targeted preemptive therapy according to perceived risk of CMV infection after kidney transplantation. Braz J Infect Dis. 2016. 10.1016/j.bjid.2016.08.007 27643978 PMC9427657

[14] Serrano OK, Matas AJ. Retransplant Outcomes Compared With First Kidney Transplants: Important Observations Not Reported in the Scientific Registry of Transplant Recipients Annual Report. Exp Clin Transplant. 2020;18(1):48–52. 10.6002/ect.2018.0244 30806202

[15] de Paula MI, Bowring MG, Shaffer AA, Garonzik-Wang J, Bessa AB, Felipe CR, et al. Decreased incidence of acute rejection without increased incidence of cytomegalovirus (CMV) infection in kidney transplant recipients receiving rabbit anti-thymocyte globulin without CMV prophylaxis—a cohort single-center study. Transpl Int. 2021;34(2):339–52. 10.1111/tri.13800 33314321 PMC8573716

[16] de Paula MI, Bae S, Shaffer AA, Garonzik-Wang J, Felipe CR, Cristelli MP, et al. The Influence of Antithymocyte Globulin Dose on the Incidence of CMV Infection in High-Risk Kidney Transplant Recipients without Pharmacological Prophylaxis. Transplantation. 2020. 10.1097/TP.0000000000003124 31978003

[17] Wekerle T, Segev D, Lechler R, Oberbauer R. Strategies for long-term preservation of kidney graft function. Lancet. 2017;389(10084):2152–62. 10.1016/S0140-6736(17)31283-7 28561006

[18] Opelz G, Döhler B. Effect of human leukocyte antigen compatibility on kidney graft survival: comparative analysis of two decades. Transplantation. 2007;84(2):137–43. 10.1097/01.tp.0000269725.74189.b9 17667803

[19] Wehmeier C, Hönger G, Cun H, Amico P, Hirt-Minkowski P, Georgalis A, et al. Donor Specificity but Not Broadness of Sensitization Is Associated With Antibody-Mediated Rejection and Graft Loss in Renal Allograft Recipients. Am J Transplant. 2017;17(8):2092–102. 10.1111/ajt.14247 28245084

[20] Ghyselen L, Naesens M. Indications, risks and impact of failed allograft nephrectomy. Transplant Rev (Orlando). 2019;33(1):48–54. 10.1016/j.trre.2018.08.001 30236837

[21] Kho MM, Bouvy AP, Cadogan M, Kraaijeveld R, Baan CC, Weimar W. The effect of low and ultra-low dosages Thymoglobulin on peripheral T, B and NK cells in kidney transplant recipients. Transpl Immunol. 2012;26(4):186–90. 10.1016/j.trim.2012.02.003 22410573

[22] Brennan DC, Daller JA, Lake KD, Cibrik D, Del Castillo D. Rabbit antithymocyte globulin versus basiliximab in renal transplantation. N Engl J Med. 2006;355(19):1967–77. 10.1056/NEJMoa060068 17093248

[23] Bachelet T, Martinez C, Del Bello A, Couzi L, Kejji S, Guidicelli G, et al. Deleterious Impact of Donor-Specific Anti-HLA Antibodies Toward HLA-Cw and HLA-DP in Kidney Transplantation. Transplantation. 2016;100(1):159–66. 10.1097/TP.0000000000000821 26262501

[24] Koch M, Kantas A, Ramcke K, Drabik AI, Nashan B. Surgical complications after kidney transplantation: different impacts of immunosuppression, graft function, patient variables, and surgical performance. Clin Transplant. 2015;29(3):252–60. 10.1111/ctr.12513 25598053

[25] de Sandes-Freitas TV, Felipe CR, Aguiar WF, Cristelli MP, Tedesco-Silva H, Medina-Pestana JO. Prolonged Delayed Graft Function Is Associated with Inferior Patient and Kidney Allograft Survivals. PLoS One. 2015;10(12):e0144188. 10.1371/journal.pone.0144188 26679933 PMC4683001

[26] Haller J, Wehmeier C, Hönger G, Hirt-Minkowski P, Gürke L, Wolff T, et al. Differential impact of delayed graft function in deceased donor renal transplant recipients with and without donor-specific HLA-antibodies. Transplantation. 2019. 10.1097/TP.0000000000002802 31205266

[27] Rincon Cintra da Cruz P, Cabral Dias Filho A, Santana V, Biela Boaretto RB, Zanettini Riccetto CL. Donor Age Amplifies the Detrimental Effects of Cold Ischemia Time on Long-Term Kidney Allograft Survival Independently of the Occurrence of Delayed Graft Function or Early Acute Rejection. Exp Clin Transplant. 2020;18(4):436–43. 10.6002/ect.2020.0066 32552628

[28] Zhou JY, Cheng J, Huang HF, Shen Y, Jiang Y, Chen JH. The effect of donor-recipient gender mismatch on short- and long-term graft survival in kidney transplantation: a systematic review and meta-analysis. Clin Transplant. 2013;27(5):764–71. 10.1111/ctr.12191 23879398

[29] Ashby VB, Leichtman AB, Rees MA, Song PX, Bray M, Wang W, et al. A Kidney Graft Survival Calculator that Accounts for Mismatches in Age, Sex, HLA, and Body Size. Clin J Am Soc Nephrol. 2017;12(7):1148–60. 10.2215/CJN.09330916 28596416 PMC5498352

[30] Guirado L. Does Rabbit Antithymocyte Globulin (Thymoglobuline®) Have a Role in Avoiding Delayed Graft Function in the Modern Era of Kidney Transplantation? J Transplant. 2018;2018:4524837. 10.1155/2018/4524837 30112193 PMC6077603

[31] Tambur AR. HLA-DQ antibodies: are they real? Are they relevant? Why so many? Curr Opin Organ Transplant. 2016;21(4):441–6. 10.1097/MOT.0000000000000325 27200497

[32] Brokhof MM, Sollinger HW, Hager DR, Muth BL, Pirsch JD, Fernandez LA, et al. Antithymocyte globulin is associated with a lower incidence of de novo donor-specific antibodies in moderately sensitized renal transplant recipients. Transplantation. 2014;97(6):612–7. 10.1097/TP.0000000000000031 24531846 PMC4106033

[33] Pascual J, Zuckermann A, Djamali A, Hertig A, Naesens M. Rabbit antithymocyte globulin and donor-specific antibodies in kidney transplantation—A review. Transplant Rev (Orlando). 2016;30(2):85–91. 10.1016/j.trre.2015.12.002 26951711

