# Supplementary figures and images for: The influence of the antithymocyte globulin dose on clinical outcomes of patients undergoing kidney retransplantation
Source: PLoS One. 2021 May 12;16(5):e0251384. doi: 10.1371/journal.pone.0251384 (PMC8115839; doi:10.1371/journal.pone.0251384)

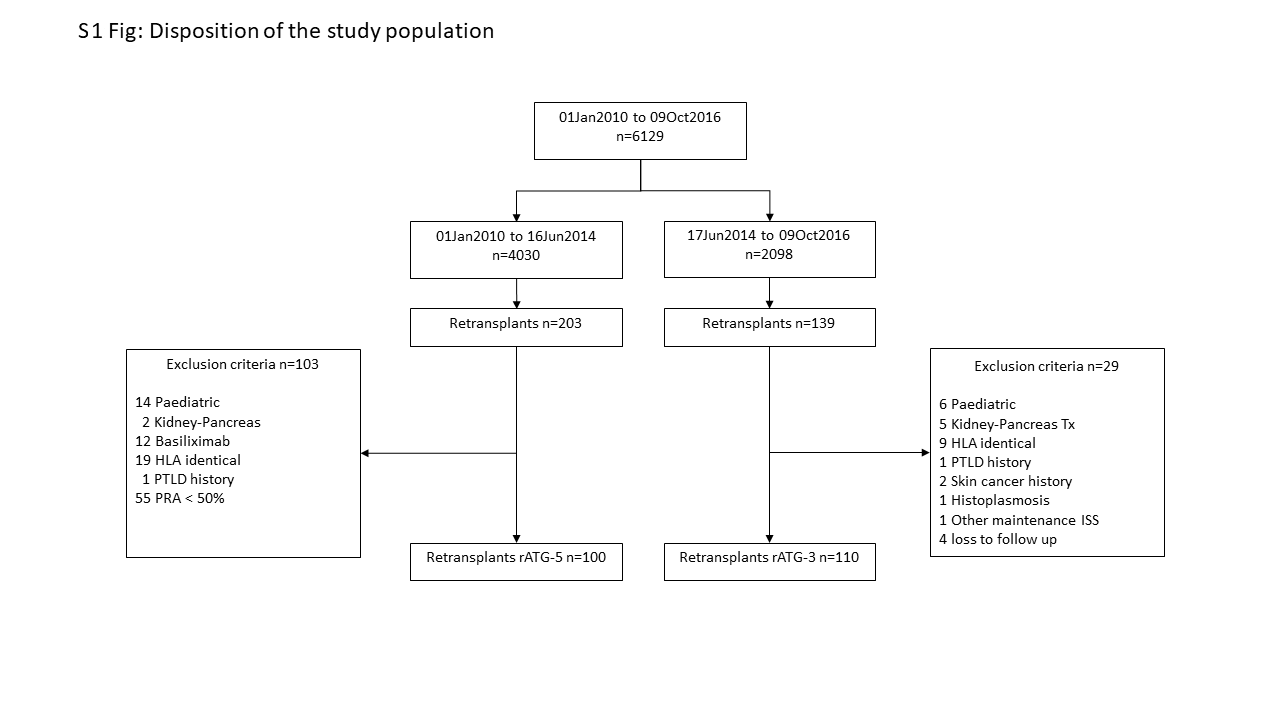

Supplement: S1 Fig — (TIF) [file pone.0251384.s001.tif]

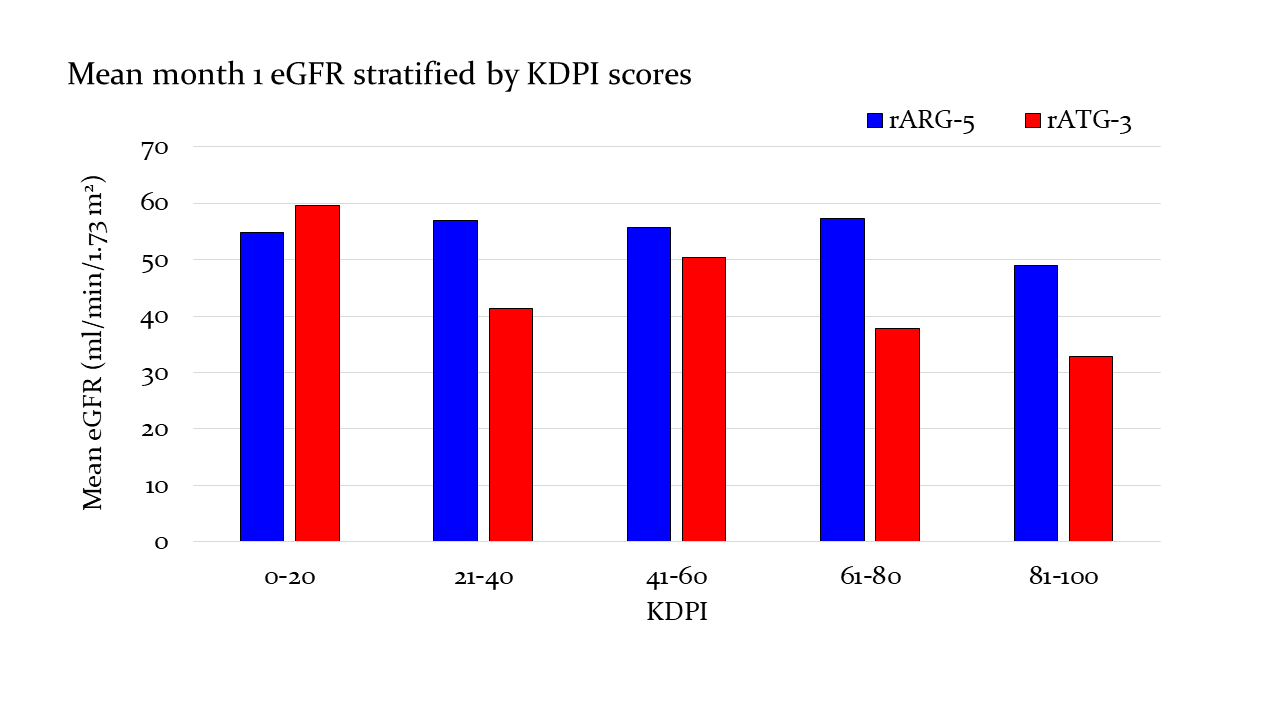

Supplement: S2 Fig — (TIF) [file pone.0251384.s002.TIF]
