# Supplementary material for: The influence of the antithymocyte globulin dose on clinical outcomes of patients undergoing kidney retransplantation
Source: PLoS One. 2021 May 12;16(5):e0251384. doi: 10.1371/journal.pone.0251384 (PMC8115839; doi:10.1371/journal.pone.0251384)
Supplement: S1 Table — (DOCX) [file pone.0251384.s003.docx]

| < 3 mg/kg  (n=4) | ≥3 to < 4 mg/kg (n=11) | ≥4 to < 5 mg/kg (n=21) | ≥5 to < 6 mg/kg (n=31) | ≥6 to < 7 mg/kg (n=13) | ≥7 to < 8 mg/kg (n=7) | ≥8 to < 9 mg/kg (n=9) | > 9 mg/kg  (n=3) |
| --- | --- | --- | --- | --- | --- | --- | --- |
| 1.3 | 3.0 | 4.0 | 5.0 | 6.0 | 7.0 | 8.0 | 9.3 |
| 1.6 | 3.1 | 4.1 | 5.0 | 6.0 | 7.1 | 8.1 | 9.8 |
| 2.3 | 3.2 | 4.1 | 5.0 | 6.1 | 7.2 | 8.1 | 10.5 |
| 2.6 | 3.2 | 4.2 | 5.0 | 6.1 | 7.3 | 8.2 |  |
|  | 3.4 | 4.2 | 5.0 | 6.1 | 7.4 | 8.3 |  |
|  | 3.5 | 4.3 | 5.0 | 6.1 | 7.5 | 8.6 |  |
|  | 3.6 | 4.4 | 5.1 | 6.2 | 7.5 | 8.8 |  |
|  | 3.7 | 4.4 | 5.1 | 6.3 |  | 8.9 |  |
|  | 3.8 | 4.4 | 5.2 | 6.4 |  | 8.9 |  |
|  | 3.8 | 4.5 | 5.2 | 6.4 |  |  |  |
|  | 3.9 | 4.5 | 5.3 | 6.5 |  |  |  |
|  |  | 4.5 | 5.3 | 6.5 |  |  |  |
|  |  | 4.6 | 5.3 | 6.9 |  |  |  |
|  |  | 4.6 | 5.3 |  |  |  |  |
|  |  | 4.6 | 5.3 |  |  |  |  |
|  |  | 4.7 | 5.4 |  |  |  |  |
|  |  | 4.7 | 5.6 |  |  |  |  |
|  |  | 4.8 | 5.6 |  |  |  |  |
|  |  | 4.8 | 5.6 |  |  |  |  |
|  |  | 4.9 | 5.6 |  |  |  |  |
|  |  | 4.9 | 5.6 |  |  |  |  |
|  |  |  | 5.6 |  |  |  |  |
|  |  |  | 5.7 |  |  |  |  |
|  |  |  | 5.7 |  |  |  |  |
|  |  |  | 5.7 |  |  |  |  |
|  |  |  | 5.8 |  |  |  |  |
|  |  |  | 5.8 |  |  |  |  |
|  |  |  | 5.8 |  |  |  |  |

S1 Table. Distribution of rATG doses in the rATG-5 group.
