# Supplementary material for: The influence of the antithymocyte globulin dose on clinical outcomes of patients undergoing kidney retransplantation
Source: PLoS One. 2021 May 12;16(5):e0251384. doi: 10.1371/journal.pone.0251384 (PMC8115839; doi:10.1371/journal.pone.0251384)
Supplement: S2 Table — (DOCX) [file pone.0251384.s004.docx]

S2 Table. Acute rejection, graft loss and deaths from 12 to 36 months.

| **Parameters, n (%)** | **rATG-5 (n=76)** | **rATG-3 (n=88)** |
| --- | --- | --- |
| Treated acute rejection episodes | 5 (6.6) | 2 (2.2) |
| *Borderline* | 1 (1.3) | 1 (1.1) |
| *IA* | 0 (0) | 0 (0) |
| *IB* | 1 (1.3) | 1 (1.1) |
| *IIA* | 1 (1.3) | 0 (0) |
| *ABMR* | 0 (0) | 0 (0) |
| *Mixed* | 0 (0) | 0 (0) |
| Clinical acute rejection | 2 (2.6) | 0 (0) |
| Graft loss | 8 (8) | 1o (9) |
| *IF/TA immune* | 3 (3.9) | 4 (4.5) |
| *IF/TA non-immune* | 1 (1.3) | 4 (4.5) |
| *Acute rejection* | 3 (3.9) | 1 (1.1) |
| *Poliomavirus nephropathy* | 1 (1.3) | 0 (0) |
| Death | 4 (5.3) | 1 (1.1) |
| *Hepatitis* | 1 (1.3) | 0 (0) |
| *Malignancy* | 1 (1.3) | 0 (0) |
| *Traffic Accident* | 1 (1.3) | 0 (0) |
| *Unknown* | 1 (1.3) | 1 (1.1) |
|  |  |  |
|  |  |  |
|  |  |  |
|  |  |  |
