# Supplementary material for: The influence of the antithymocyte globulin dose on clinical outcomes of patients undergoing kidney retransplantation
Source: PLoS One. 2021 May 12;16(5):e0251384. doi: 10.1371/journal.pone.0251384 (PMC8115839; doi:10.1371/journal.pone.0251384)
Supplement: S3 Table — (DOCX) [file pone.0251384.s005.docx]

S3 Table. Trajectories of kidney function stratified by the presence of treated acute rejection (tAR).

| **Parameters** | **rATG-5 (n=100)** | **rATG-3 (n=110)** | **p value** |
| --- | --- | --- | --- |
| No tAR, n (%) | 77 (77) | 90 (81.8) | p=0.597 |
| eGFR, mL/min ± SD |  |  |  |
| *Month 1* | 56.96 ± 29.85 | 44.96 ± 25.60 | p=0.007 |
| *Month 3* | 61.31 ± 27.50 | 47.84 ± 25.33 | p=0.001 |
| *Month 6* | 60.97 ±27.20 | 48.18 ± 25.64 | p=0.001 |
| *Month 12* | 59.37 ± 27.75 | 47.57 ± 25.18 | p=0.001 |
| *Month 24* | 53.58 ± 28.39 | 39.73 ± 24.14 | p=0.019 |
| *Month 36* | 53.25 ± 28.22 | 44.58 ± 24.00 | p=0.019 |
| tAR, n (%) | 23 (23) | 20 (18.2) | p=0.597 |
| eGFR, mL/min ± SD |  |  |  |
| *Month 1* | 47.61 ± 23.95 | 38.77 ± 25.22 | p=0.271 |
| *Month 3* | 48.21 ± 25.71 | 41.09 ± 21.84 | p=0.286 |
| *Month 6* | 47.09 ± 25.96 | 42.72 ± 23.62 | p=0.375 |
| *Month 12* | 46.61 ± 25.06 | 35.86 ± 27.18 | p=0.082 |
| *Month 24* | 43.90 ± 25.47 | 34.46 ± 25.61 | p=0.125 |
| *Month 36* | 43.69 ± 28.22 | 34.22 ± 25.42 | p=0.125 |
