# Supplementary material for: The influence of the antithymocyte globulin dose on clinical outcomes of patients undergoing kidney retransplantation
Source: PLoS One. 2021 May 12;16(5):e0251384. doi: 10.1371/journal.pone.0251384 (PMC8115839; doi:10.1371/journal.pone.0251384)
Supplement: S4 Table — (DOCX) [file pone.0251384.s006.docx]

S4 Table. Renal function stratified by donor and recipient gender combinations.

| **Parameters** | **rATG-5 (n=100)** | | **rATG-3 (n=110)** | | **p value** |
| --- | --- | --- | --- | --- | --- |
| eGFR (ml/min/1.73 m^2^, month 1) |  |  |  |  |  |
| *Donor male/recipient male* | 34 (34.7) | 53.8±28.0 | 36 (33.3) | 41.4±25.0 | 0.028 |
| *Donor female/recipient male* | 18 (18.4) | 51.4±29.6 | 30 (27.8) | 52.9±23.7 | 0.850 |
| *Donor male/recipient female* | 30 (30.6) | 58.6±32.6 | 17 (15.7) | 40.9±25.7 | 0.061 |
| *Donor female/recipient female* | 16 (16.3) | 59.2±17.0 | 25 (23.1) | 43.4±24.0 | 0.055 |
| eGFR (ml/min/1.73 m^2^, month 12) |  |  |  |  |  |
| *Donor male/recipient male* | 33 (37.5) | 60.2±22.2.9 | 31 (32.3) | 52.7±21.9 | 0.180 |
| *Donor female/recipient male* | 14 (15.9) | 62.9±20.8 | 26 (27.1) | 52.8±21.6 | 0.163 |
| *Donor male/recipient female* | 25 (28.4) | 69.2±17.7 | 15 (15.6) | 44.8±21.4 | 0.000 |
| *Donor female/recipient female* | 16 (18.2) | 63.4±21.8 | 24 (25) | 45.3±19.5 | 0.009 |
