# Supplementary material for: The influence of the antithymocyte globulin dose on clinical outcomes of patients undergoing kidney retransplantation
Source: PLoS One. 2021 May 12;16(5):e0251384. doi: 10.1371/journal.pone.0251384 (PMC8115839; doi:10.1371/journal.pone.0251384)
Supplement: S1 Appendix — (DOCX) [file pone.0251384.s007.docx]

# INFORMED CONSENT FORM

**SOLID ORGANS AND TISSUES DONATION – DECEASED DONOR – OVER 18 YEARS-OLD**

## According the local regulation and laws 9.434/97 and 10.211/01, I authorize the solid organs and/or tissues of:

Donor: Social Security Number: Date of Birth: / / Age: Naturality: State/Province: Address: City: , State/Province: Zipcode: _

**RESPONSIBLE FOR AUTHORIZATION**

***Relationship: ( ) Father ( ) Mother ( ) Grandparent ( ) Sibling ( ) Spouse ( ) Offspring**

**( ) Grandchild ( ) Legal Responsible ( ) Proven judicial authorization**

Name: Naturality: State/Province: Social Security Number: Address: : City: State/Province: Zipcode Telephone: Signature: Date: / /

**I authorize the donation of organs:** (heart, lungs, liver, pancreas, kidneys, intestine) and tissues (corneas, osteotendinous, skin, vessels, heart valves)

( ) Exclusively for transplantation

( ) Organ or Tissue that is not used for transplantation, due to its quality, may be used for scientific purposes (Teaching/Research)

**Note:** The authorizer **"does not accept"** the removal, specifically, of the Organ/tissue

**WITNESSES**

1. **Name** Social Secutiry Number: Telephone: Address: Signature:
2. **Name**

Social Security Number: Telephone: Address: Signature:

Location e Date: , Time:

**(Professional responsible for family interview (stamp and signature and professional registration)**
