# Supplementary material for: The influence of the antithymocyte globulin dose on clinical outcomes of patients undergoing kidney retransplantation
Source: PLoS One. 2021 May 12;16(5):e0251384. doi: 10.1371/journal.pone.0251384 (PMC8115839; doi:10.1371/journal.pone.0251384)
Supplement: S2 Appendix — (DOCX) [file pone.0251384.s008.docx]

TERM OF AUTHORIZATION FOR KIDNEY DONATION FROM TRANSPLANTATION

|  |  | **São Paulo, / /** |
| --- | --- | --- |
| I, , Social Secutiry Number_______________ resident of ___________ agree, accordance with Article 9, Chapter III of the Law of  9,434 of 4 February 1997, as amended by Law No. 10,211 of March 23, 2001, to donate one kidney to Mr(s)/Miss  __________________________________________, Social Security Number___________ . There is a relationship between us and I am _________________________ of the recipient.  I am aware that:   1. Kidney transplantation with a living donor consists of the removal of a kidney from a healthy donor and implantation of this organ in a patient with chronic kidney disease. The donation is a spontaneous, selfless act and will not bring any direct benefit to me. I agreed to perform it because of my family and affective connection with the receiver. Kidney donation does not interfere with my social and professional activities. 2. This surgery has a small mortality risk of 3 deaths per 10,000 surgeries. Transient or definitive impairment of my health may occur, which may lead to temporary or permanent physical disability. 3. Although it is a rare event the kidney I am donating may be rejected or thrombosed and, if necessary, removed from the recipient when there is no longer a chance of recovery. 4. I did a complete preoperative evaluation, with laboratory, imaging and ABO and HLA compatibility tests 5. Anesthetic, surgical, infectious and cardiovascular complications are rare and occur on less than 1% of occasions. 6. The average length of hospital stay for the donation is 3 days. However, if anesthetic, surgical, infectious or cardiovascular complications occur, it may be necessary to extend the hospitalization period, including in the intensive care unit (ICU). 7. I received guidance regarding the risks to my health of being obese, smoking and hypertensive, before and after kidney donation. 8. The use of illicit drugs may contraindicate kidney donation for my safety and preservation of my health. 9. Renal functions can be performed in a normal way by a single kidney, but it is extremely important to do the annual medical follow-up for evaluation of my health, including blood pressure control and laboratory tests to assess renal function and other possible changes related to donation. 10. I declared that all the information provided up to that moment is true, aware that the transplant may be suspended if there is a change in the tests or divergence in the information provided by me. | | |
| After reading this term, I declare that Doctor ________________________________________Medical Registration: __________ , clarified to all the questions I presented in relation to the proposed surgery and agree with its performance. My decision to donate organ is being communicated to the Public Ministry. | | |
|  | DONOR’S SIGNATURE |  |
| WITNESSES: | | |
| Name: |  | Name: |
| Social Secutiry Number: |  | Social Security Number: |

364 – (03/20)
