# Supplementary material for: The influence of the antithymocyte globulin dose on clinical outcomes of patients undergoing kidney retransplantation
Source: PLoS One. 2021 May 12;16(5):e0251384. doi: 10.1371/journal.pone.0251384 (PMC8115839; doi:10.1371/journal.pone.0251384)
Supplement: Attachment — Submitted filename: Response to Reviewers.docx [file pone.0251384.s010.docx]

Dear Justyna Gołębiewska
Academic Editor
PLOS ONE

Thank you the comments and suggestion provide to our manuscript

PONE-D-21-00777: The influence of the Antithymocyte Globulin dose on clinical outcomes of patients undergoing kidney retransplantation. Please find below a point-by-point reply to each of the editors’ and reviewers’ comments.

Editor

This manuscript is of interest to the renal transplant community. However, it is not acceptable for publication in its current form.

The manuscript requires more detailed methodology and results description as pointed out by both Reviewers, especially:

1. The power of the study to detect a difference in the primary endpoint should be stated, or are the author’s wanting to demonstrate non-inferiority.

YES! The aim was to demonstrated non-inferiority. This was added to the methods section (see below).

2. The study population, baseline characteristics and outcome assessment is unclear as described in detail by Reviewer 3.

We clarified all issues raised by the reviewers regarding study population, baseline characteristics and outcome assessments (see below).

3. It is unclear what dose the 5 mg/kg group actually received because doses were increased or decreased on changes in lymphocyte count after each dose. The actual dose given should be reported.

YES. We now provide the actual doses given (see below).

4. Please explain the details of loss to follow-up, e.g., reasons and causes. How was loss to follow up handled in analyses?

We explained the details of loss to follow-up and how was it was handled in the analyses.

5. The much higher biopsy rate in the 3 mg/kg group needs better explanation.

Detailed explanation regarding the higher biopsy rate in the 3 mg/kg group is provided below.

If applicable, we recommend that you deposit your laboratory protocols in protocols.io to enhance the reproducibility of your results. Protocols.io assigns your protocol its own identifier (DOI) so that it can be cited independently in the future. For instructions see: <http://journals.plos.org/plosone/s/submission-guidelines#loc-laboratory-protocols>

**Journal Requirements:**

When submitting your revision, we need you to address these additional requirements.

1. Please ensure that your manuscript meets PLOS ONE's style requirements, including those for file naming. The PLOS ONE style templates can be found at

https://journals.plos.org/plosone/s/file?id=wjVg/PLOSOne_formatting_sample_main_body.pdf and

[https://journals.plos.org/plosone/s/file?id=ba62/PLOSOne_formatting_sample_title_authors_affiliations.pdf](about:blank)

We formatted our manuscript according to the PLOS ONE style requirements.

2. In your ethics statement in the manuscript and in the online submission form, please ensure that you have discussed whether all data/samples were fully anonymized before you accessed them and/or whether the IRB or ethics committee waived the requirement for informed consent.

The research project was approved by the local Research Ethics Committee of the Federal University of São Paulo (CAAE 02285018.2.5505). The IRB waived the requirement to obtain informed consent because of the retrospective nature of the record review, the lack of interference with the rights and welfare, and the lack of risk to the participants. All data were retrieved from the electronic medical records and were fully anonymized before analysis. Medical records of the Hospital do Rim (Fundação Oswaldo Ramos) were accessed between 01/06/2017 and 01/10/2020.

If patients provided informed written consent to have data/samples from their medical records used in research, please include this information.

 Not applicable

3. In the ethics statement in the manuscript and in the online submission form, please provide additional information about the patient records/samples used in your retrospective study, including:

a) the date range (month and year) during which patients' medical records/samples were accessed;

Medical records were accessed between 01/06/2017 and 01/10/2020.

b) the source of the medical records/samples analyzed in this work (e.g. hospital, institution or medical center name).

 Medical records of the Hospital do Rim (fundação Oswaldo Ramos) were accessed between 01/06/2017 and 01/10/2020.

4. We note that your study involved tissue/organ transplantation. Please provide the following information regarding tissue/organ donors for transplantation cases analyzed in your study.

1. Please provide the source(s) of the transplanted tissue/organs used in the study, including the institution name and a non-identifying description of the donor(s).

Kidney transplant were performed at the Hospital do Rim with organs recovered from deceased donors under the National and São Paulo State secretary allocation policy, and from living donors, according to the National legislation.
2. Please state in your response letter and ethics statement whether the transplant cases for this study involved any vulnerable populations; for example, tissue/organs from prisoners, subjects with reduced mental capacity due to illness or age, or minors.
- If a vulnerable population was used, please describe the population, justify the decision to use tissue/organ donations from this group, and clearly describe what measures were taken in the informed consent procedure to assure protection of the vulnerable group and avoid coercion.
- If a vulnerable population was not used, please state in your ethics statement, “None of the transplant donors was from a vulnerable population and all donors or next of kin provided written informed consent that was freely given.”
3. In the Methods, please provide detailed information about the procedure by which informed consent was obtained from organ/tissue donors or their next of kin. In addition, please provide a blank example of the form used to obtain consent from donors, and an English translation if the original is in a different language.

Informed consent was obtained from all donors or their next of kin, prior to organ recovery. Translated informed consent forms for deceased and living donors are now provided as supplemental material.
4. Please indicate whether the donors were previously registered as organ donors. If tissues/organs were obtained from deceased donors or cadavers, please provide details as to the donors’ cause(s) of death.

The national regulation does not mandate registration of donors. The deceased donors causes of death are provided in Table 1.
5. Please provide the participant recruitment dates and the period during which transplant procedures were done (as month and year).

Record data from kidney transplants performed between 01Jan2010 to 09Oct2016 were screened for this analysis.
6. Please discuss whether medical costs were covered or other cash payments were provided to the family of the donor. If so, please specify the value of this support (in local currency and equivalent to U.S. dollars).

According to Brazilian legislation, no medical costs or other cash payments are allowed to the family of the donor.

5. We note that you have indicated that data from this study are available upon request. PLOS only allows data to be available upon request if there are legal or ethical restrictions on sharing data publicly. For information on unacceptable data access restrictions, please see http://journals.plos.org/plosone/s/data-availability#loc-unacceptable-data-access-restrictions.

In your revised cover letter, please address the following prompts:

a) If there are ethical or legal restrictions on sharing a de-identified data set, please explain them in detail (e.g., data contain potentially identifying or sensitive patient information) and who has imposed them (e.g., an ethics committee). Please also provide contact information for a data access committee, ethics committee, or other institutional body to which data requests may be sent.

There are no restrictions

b) If there are no restrictions, please upload the minimal anonymized data set necessary to replicate your study findings as either Supporting Information files or to a stable, public repository and provide us with the relevant URLs, DOIs, or accession numbers. Please see http://www.bmj.com/content/340/bmj.c181.long for guidelines on how to de-identify and prepare clinical data for publication. For a list of acceptable repositories, please see http://journals.plos.org/plosone/s/data-availability#loc-recommended-repositories.

The anonymized data set necessary to replicate our study findings is provided as S3-Appendix-Master file.

We will update your Data Availability statement on your behalf to reflect the information you provide.

6. Thank you for stating the following in the Competing Interests section:

'I have read the journal's policy and the authors of this manuscript have the following competing interests:

Helio Tedesco-Silva has received speaker’s fees and travel or accommodation expenses for development of educational presentations and scientific advice from Novartis, Pfizer, and Roche.
Jose Medina Pestana has received speaker’s fees and travel or accommodation expenses for development of educational presentations and scientific advice from Bristol-Myers Squibb, Novartis, Pfizer, and Roche.
Marina Pontello Cristelli has received speaker’s fees for development of educational presentations and travel or accommodation expenses from Novartis and Pfizer.
The other authors of this manuscript have no conflicts of interest to disclose.'

a. Please confirm that this does not alter your adherence to all PLOS ONE policies on sharing data and materials, by including the following statement: "This does not alter our adherence to PLOS ONE policies on sharing data and materials.”

(as detailed online in our guide for authors [http://journals.plos.org/plosone/s/competing-interests](about:blank)).  If there are restrictions on sharing of data and/or materials, please state these.

Please note that we cannot proceed with consideration of your article until this information has been declared.

b. Please include your updated Competing Interests statement in your cover letter; we will change the online submission form on your behalf.

There is no new information to update in the Competing Interests statement.

Please know it is PLOS ONE policy for corresponding authors to declare, on behalf of all authors, all potential competing interests for the purposes of transparency. PLOS defines a competing interest as anything that interferes with, or could reasonably be perceived as interfering with, the full and objective presentation, peer review, editorial decision-making, or publication of research or non-research articles submitted to one of the journals. Competing interests can be financial or non-financial, professional, or personal. Competing interests can arise in relationship to an organization or another person. Please follow this link to our website for more details on competing interests: [http://journals.plos.org/plosone/s/competing-interests](about:blank)

[Note: HTML markup is below. Please do not edit.]

Reviewers' comments:

Reviewer's Responses to Questions

**Comments to the Author**

1. Is the manuscript technically sound, and do the data support the conclusions?

The manuscript must describe a technically sound piece of scientific research with data that supports the conclusions. Experiments must have been conducted rigorously, with appropriate controls, replication, and sample sizes. The conclusions must be drawn appropriately based on the data presented.

Reviewer #1: Yes

Reviewer #3: Partly

2. Has the statistical analysis been performed appropriately and rigorously?

Reviewer #1: Yes

Reviewer #3: Yes

3. Have the authors made all data underlying the findings in their manuscript fully available?

The [PLOS Data policy](http://www.plosone.org/static/policies.action#sharing) requires authors to make all data underlying the findings described in their manuscript fully available without restriction, with rare exception (please refer to the Data Availability Statement in the manuscript PDF file). The data should be provided as part of the manuscript or its supporting information, or deposited to a public repository. For example, in addition to summary statistics, the data points behind means, medians and variance measures should be available. If there are restrictions on publicly sharing data—e.g. participant privacy or use of data from a third party—those must be specified.

Reviewer #1: Yes

Reviewer #3: Yes

4. Is the manuscript presented in an intelligible fashion and written in standard English?

PLOS ONE does not copyedit accepted manuscripts, so the language in submitted articles must be clear, correct, and unambiguous. Any typographical or grammatical errors should be corrected at revision, so please note any specific errors here.

Reviewer #1: Yes

Reviewer #3: Yes

5. Review Comments to the Author

Please use the space provided to explain your answers to the questions above. You may also include additional comments for the author, including concerns about dual publication, research ethics, or publication ethics. (Please upload your review as an attachment if it exceeds 20,000 characters)

Reviewer #1: This study examines the influence of the Antithymocyte Globulin dose on clinical outcomes of patients undergoing kidney retransplantation. The authors compared the efficacy and safety of rATG-5 and rATG-3. The outcomes of kidney transplantation were evaluated comprehensively. However, the research question seems not timely as the FDA has approved rATG as an induction drug with typical dose recommendations of 1.5 mg/kg in 2018. Studies have already compared efficacy and safety between rATG-3 with rATG-1.5. Perhaps it isn't an urgent topic now to compare rATG-5 to rATG-3. Below are my minor comments about study design and statistical analysis:

While the FDA approved the use of rATG for induction therapy, the dosing regimen is still open for debate, depending on the immunological risk and the risk of developing delayed graft function. Our main goal was to demonstrate that the use of a single 3 mg/kg dose of rATG would provide comparable efficacy to the traditional 5 mg/kg dose, in high immunological risk patients undergoing retransplantation but without pre-formed HLA A, B, DR donor specific antibodies.

1. Authors mentioned that this is a natural experiental study. However, time bias might be induced by following two comparison groups in different time periods.

YES! We changed the study design as a sequential cohort study.
2. Ethical approval for this study should be mentioned in the data source section.

Ethical approval for this study is reported in the Ethics section.
3. Lack of statistical significance for comparisons could be due to the rate events in two groups. Please calculate the statistical power for the major outcomes and discuss it in limitations.

YES! The sample size lack sufficient statistical power considering the significance event rate events in the two groups. In fact, this was a non-inferiority trial and detailed of the sample size and power were now added. The following paragraph was added to the statistical analysis section.

“Considering the non-inferiority margin of 15% for the experimental rATG-3 group compared to the standard rATG-5 group for the primary endpoint of efficacy failure at 12 months, a significance level of 5%, a statistical power of 80%, and the efficacy failure of 25% in the control arm, 208 patients are required to be 80% sure that the upper limit of a one-sided 95% confidence interval. We used Cox proportional hazard model to compare hazard functions using treatment group as factor. Non-inferiority was determined if upper confidence limit of HR was less than the non-inferiority margin then non-inferiority using Wald’s confidence interval.”
4. Please explain the details of loss to follow-up, e.g., reasons and causes. How was loss to follow up handled in analyses?

The only cause of loss to follow up was referral to another transplant center. For survival analysis, patients with loss to follow up were censored. For comparison of the trajectories of renal function, last observation carried forward analysis was used.
5. Table 6 is only for 1 month eGFR < 48 ml/min/1.73 m2. The interaction of rATG and time can be examined in the model, then check the differences of eGFR by rATG at each time point.

Apologies for our lack of clarity. Table 6 includes all patients in both groups (n=210) and we performed a multivariable logistic regression to identify the risk factors associated with reaching 1 month with an eGFR < 48 ml/min/1.73 m^2^. We believe this is the most appropriate statistical approach. In addition, as shown in Figure 3, the trajectories of renal function are parallel from month. Therefore, differences of eGFR at each time point persisted throughout the first year.

Reviewer #3: Overall: The study addresses an issue for which there is limited high quality data. It suffers from the usual limitations of retrospective studies. The power of the study to detect a difference in the primary endpoint should be stated, or are the author’s wanting to demonstrate non-inferiority – this should be clarified. There a number of clarifications/corrections needed -see details below. I think the study despite its limitations will be of some interest to readers, but I do not think the retrospective data will change practice. The much higher biopsy rate in the 3 mg/kg group needs better explanation, as does this higher rate of biopsy would like negate any economic benefit of the lower dosing.
YES! The sample size lack sufficient statistical power considering the event rate in the two groups. In fact, this was a non-inferiority trial and detailed of the sample size and power were now added.

We provided several evidences suggesting that the higher rate of biopsies in the 3 mg/kg group was associated with donor characteristics (see below), although. The perceived benefits of the lower dose are (1) improved dosing schedule: only one dose within the first 24 hours after graft revascularization; (2) improved safety monitoring: during the infusion that patients are in an intensive monitoring unit; (3) earlier hospital discharge before day 5; (4) shorter duration of lymphopenia (unfortunately this was not investigated in this study – see reference 18); (5) increased safety, not seen in this analysis, but observed in another high-risk population using the same strategy (reference 19).

Significance: There is little information about alternative dosing strategies for T cell depleting therapy. It is unlikely that a RCT will be conducted and registry data do not include dosing information -therefore single or multi-center retrospective series are likely to be the best available source of information.
We fully agree with the reviewer. We decided to use universal 3 mg/kg rATG induction therapy, except for recipients of HLA identical living donor kidney transplants, after an initial data obtained from a prospective randomized trial (reference 12). We then allowed sufficient time to gather minimum data suggesting that this strategy is effective and safe in low-risk (reference 15) and high-risk (reference 16) kidney transplant recipients. We are now describing the initial experience with retransplants. Altogether these data suggests that this strategy is feasible but, of course, cannot be immediately extrapolated to other populations without careful monitoring.
Background Suggested Improvements:

As outlined in the introduction, retransplant patients are known to have worse outcomes than first transplant recipients and are considered to be at increased immunological risk. The authors may want to consider expanding on the reasons behind the increased immunological risk (intro or discussion) highlighting the potential roles of increased sensitisation and repeat class 2 HLA mismatches (e.g. Tinkham et al 2016). They may also consider providing data on the increasing incidence of retransplant as a proportion of all transplants undertaken (to support the relevance of this study) and also briefly summarise previous studies that have investigated induction regimens in retransplant recipients (e.g. Schold et al, 2015), and moreover outcomes with different ATG dosing regimens in transplantation in general (e.g singh et al, 2018). I feel having this information within the manuscript somewhere will give the reader greater context for the current study.
We agree with the reviewer. We included these data in the introduction and added the suggested references.
The current study takes advantage of a change in policy in a large single center and compares the standard of care (SOC 5X 1mg/kg dosing used in the period Jan 2010- Jun 2014) versus a novel single 3 mg / kg dose (used in the period June 2014 – Oct 16) in retransplant recipients of a living or deceased donor transplant

The key finding was no difference in the composite efficacy outcome of biopsy proven acute rejection, death or graft loss and no difference in safety indicators including 30 day re-hospitalization, CMV infection/disease.

There were 100 / 110 patients in each group with 3 year post transplant outcomes reported.

Although this is a retrospective study – it is not clear what the type II error is in this relatively small study cohort.
YES! The sample size lack sufficient statistical power considering the event rate in the two groups. In fact, this was a non-inferiority trial and detailed of the sample size and power were now added. This limitation was highlighted in the discussion.
In addition to the usual limitations of retrospective studies there are a number of clarifications required to assess this study:

1. The study population is unclear: a) The number transplanted – number excluded does not add up to the number included in the study.

YES! The numbers were not accurate. Please see below the correction. We also included a Supplemental Figure 1 with the disposition of the study population.

“There were 4030 kidney transplants between January 1, 2010 and June 16, 2014, of which 203 (5%) were retransplants, while among 2098 procedures between June 17, 2014 and October 9, 2016, 139 (6.6%) were retransplants. We excluded 103 retransplants in the first period and 29 in the second period who did not receive induction therapy with rATG (S4-Figure 1).”

2) The study protocol states that study patients in the 1 mg/kg group all had cPRA >50% - but there are clearly patients in this group with cPRA <50%.

YES! This was not clear. cPRA was in fact calculated retrospectively for the comparison between the two groups in this analysis. Our institutional protocol used 1 mg/kg of rATG for patients with PRA class I or class II > 50%.

While all recipients with PRA>50% received up to 5 doses of 1 mg/kg of rATG, patients with PRA< 50% could receive the same induction strategy based on other risk factors such as HLA compatibility, cold ischemia time, priority criterion, and donor type. The proportion of patients with PRA class I or class II >50% was 65% (n=65) in the rATG-5 group and 56% (n=62) in the rATG-3 group. This information was added to the methods and result sections.

The method of assessment for DSA should be provided.

To identify anti-HLA antibodies in serum, FlowPRA screening test is performed, followed by single antigen bead assay to identify the specificity of anti-HLA antibodies, using the Luminex platform.

2. Baseline characteristics – it is not clear what initial and final Cr and delta Cr mean in Table 1.

Initial creatinine value is the first value obtained at the time of hospital admission of the potential donor. Final creatinine is the last creatinine value before organ recovery. Delta creatinine is an arbitrary measure of acute kidney injury. This clarification was added as a footnote in Table 1.

How was KDPI determined? – against what reference population? I am not aware that Brazil uses a KDPI calculator.

KDPI was calculated using the UNOS formula developed using registry data from the North American kidney transplant population. KDPI has not been validated in Brazil as none of the other indexes developed in North America (for example, the older “extended criteria donor” definition). We used KPDI as a more granular measure of kidney donor “quality” for simple comparison between the two groups

Were there no DCD donor kidneys used?

In Brazil, the use of organ from DCD donors is forbidden by law.

3. It is unclear what dose the 5mg/kg group actually received because doses were increased or decreased on changes in lymphocyte count after each dose. The actual dose given should be reported – it may be that a significant % of those in the 5 mg/kg group actually received 3 mg/kg or even less. As lymphopenia is an important outcome, it would be important to know the incidence of this in both groups.

In the rATG 5 mg/kg group, 2 patients received 1, 3 patients received 2, 1 patient received 3, 11 patients received 4, 80 patients received 5, and 3 patients received 6 doses of rATG.

In the rATG 5 mg only 4 patients received less than 3 mg/kg, 32 received between 3 and 5 mg/kg, 32 received between 5 and 6 mg/kg, and 32 more than 6 mg/kg S5- Table 1).

4. Outcome assessment – the protocol states all biopsies for rejection were determined using the Banff 2019 criteria – this cannot be the case as most of the patients were included before 2019. What criteria were used to determine/classify AR. Was there retrospective review of all AR biopsies?
All biopsies with rejection were retrospectively reclassified using the Banff 2019 criteria.
5. Outcome assessment – what was the test platform used for CMV antigenemia determination and what were the test parameters. If these changed over time it may be better to report those requiring ganciclovir treatment.

During the study period, the CMV antigenemia test was performed in a single laboratory using the CMV Brite Turbo kit, according to the manufacturer's recommendations (IQ® Products, Groningen, Netherlands). The data provided (Table 4) refers to patients treated with ganciclovir meeting the protocol defined criteria for CMV infection or disease.
6. Outcome assessment -as there was an imbalance in Everolimus maintenance between groups – and EVR is known to be protective against CMV – was the incidence of CMV different between groups among patients treated with CNI/MPA.

None of the patients receiving EVR in both groups developed CMV infection, as well as the 3 patients who received azathioprine in the rATG-3 group.

Therefore, there was no difference incidence of CMV infection comparing patients receiving CNI/MPA in the rATG-5 (49%, 48/98) and in the raTG-3 (45%, 44/98).

7. Outcome assessment – the far higher bx rate in the 3mg/kg cohort requires explanation.
The higher rate of kidney biopsies in the 3 mg/kg dose group is a consequence of early and incomplete recovery of kidney function after transplantation, as demonstrated by the difference in GFR at month 1. Because of the perceived higher risk for acute rejection among recipients of retransplants, small increases in creatinine triggered the indication of a biopsy to rule out rejection. Despite undergoing a higher number of kidney biopsies, there was no statistical difference in the number of treated acute rejections. Finally, the analysis of the chronic Banff scores suggests that the difference in kidney function is associated, at least in part, by the chronic cg scores observed in both early and late biopsies.

8. There were more humoral rejections in the 3 mg/kg group – the authors should convince us that the lower overall level of kidney function in the 3mg/kg group is not due to this.

There are several evidences suggesting that the unbalanced rejection rates between the two groups did not influence kidney function. First, the incidence of treated rejection was low in both groups (20% rATG-5 vs. 17.3% rATg-3). Second, the difference in kidney function was noticed as early as 1 month (Figure 3), when not all rejection had occurred, and persisted throughout the follow up. Thirdly, the trajectories of kidney function in patients without treated acute rejection showed the same pattern as the overall population (S7- Table 3).

9. The authors should confirm if any/what other changes in practice occurred over this timeframe.

There were no further changes in the protocols or in clinical practice over the time frame of this study.

We note, for example, that the change in practice was based on avoiding transplantation in patients with preformed antibody to HLA A, B and DR antigens – does this mean that in the cohort prior to the change patients were being potentially transplanted in the presence of such antibodies (albeit CDC XM neg)?

In both cohorts of patients (rATG-5 and rATG-3) patients with preformed antibody to HLA A, B and DR antigens were noy eligible for kidney transplantation.

Was flow XM undertaken at any stage during the study?

No, only recently we started using flow XM.

Were any other changes undertaken during the period to improve HLA matching?

NO.

The authors state that in the first period all patients with PRA >50% received ATG whereas PRA wasn’t taken into consideration in the second period. This should be clarified as table 1 demonstrates some patients in the first period have PRA under 50%. Together, this suggests the cohorts may have been of unequal immunological risk. Please address.

YES! This was not clear. While all recipients with PRA>50% received up to 5 doses of 1 mg/kg of rATG, patients with PRA< 50% could receive the same induction strategy based on other risk factors such as HLA compatibility, cold ischemia time, priority criterion, and donor type. The proportion of patients with PRA class I or class II >50% was 65% (n=65) in the rATG-5 group and 56% (n=62) in the rATG-3 group. This information was added to the methods and result sections.
