# Supplementary material for: The influence of the antithymocyte globulin dose on clinical outcomes of patients undergoing kidney retransplantation
Source: PLoS One. 2021 May 12;16(5):e0251384. doi: 10.1371/journal.pone.0251384 (PMC8115839; doi:10.1371/journal.pone.0251384)
Supplement: Attachment — Submitted filename: Response to Reviewers - 2.docx [file pone.0251384.s011.docx]

Dear Justyna Gołębiewska
Academic Editor
PLOS ONE

Thanks for the new insights provide to our manuscript. Please find below a point-by-point clarification of the pending issues

ACADEMIC EDITOR:

Thank you for all the new information provided and for all explanations included. However, there are still issues that require clarification.

“In the rATG-5 group only 4 patients received less than 3 mg/kg, 32 received between 3 and 5 mg/kg” – 36 out of 100 patients in rATG-5 group did not receive 5mg/kg. This is a source of a substantial bias and has not even been acknowledged in the discussion. This is a retrospective analysis, not a prospective randomized clinical trial, the patients should be divided into groups according to the actual dose they received and all results recalculated. Another way would be to exclude these 36 patients from the analysis, but as I understand the study would be then underpowered to even show non-inferiority.

YES! The editor is quite correct and this has to be highlighted in our manuscript. This could be a source of a substantial bias and has not even been acknowledged in the discussion. Below we discuss this possible bias, added more data and analysis, and acknowledge it in the discussion section.

In the seminal manuscript by Brennan D et al, which we use as reference and adapted to implement our previous standard of care induction therapy “rabbit antithymocyte globulin was initiated before reperfusion in 87.9% of all patients assigned to receive it, and 68.8% received the intended five doses.” [Brennan DC, Daller JA, Lake KD, Cibrik D, Del Castillo D; Thymoglobulin Induction Study Group. Rabbit antithymocyte globulin versus basiliximab in renal transplantation. N Engl J Med. 2006 Nov 9;355(19):1967-77. doi: 10.1056/NEJMoa060068. PMID: 17093248.]

In fact, in all studies addressing alternative doses of r-ATG, the control group was the current “standard of care”. Yet, the actual doses are always lower than that described in the methods section in each manuscript, as all analyses were performed as “intention to treat”.

We added the following paragraph to the methods section:

“All comparisons were made using the intention to treat population, defined as patients receiving at least of dose of rATG in both groups.”

The reasons to reduce the intended total dose are primarily safety issues, such as leukopenia, thrombocytopenia and surgical complications. The same issue was observed when we analyzed the same strategy in high-risk kidney transplant recipients (reference 16). The debate is even expanded as the pharmacodynamic measure (lymphopenia) does not correlate with dose and is not routinely used.

Overall, it is difficult to determine whether the bias in favor or against the rATG-5 group. The inability to administer the 5 doses as scheduled could be interpreted as “treatment failure” because the limiting toxicity may increase the risk of acute rejection. The safety of the single 3 mg/kg dose approach is therefore an advantage compared to the 5 doses of 1 mg/kg regimen.

We reviewed most of these manuscripts to determine a lower r-ATG dose that could provide a balanced efficacy/safety profile and ultimately tested this 3 mg/kg r-ATG single dose in a prospective trial (reference 12) before adopting it as our new “standard of care”.

The patients should be divided into groups according to the actual dose they received and all results recalculated.

We added the following paragraph to the result section:

Because 36 patients in the rATG group did not receive the intended total dose due to adverse events, a subgroup analysis revealed a higher incidence of first treated acute rejection (30.6% vs. 18.8%, p=0.178) and first BCAR ≥ IA (19.4 vs. 4.7%, p=0.033) comparing patients receiving < 5 mg/kg (n=36) or ≥ 5 mg/kg (n=64) total dose of rATG in the rATG-5 group.

We added the following paragraph to the discussion section:

Importantly, only 64% of the patients in the rATG-5 receive the full 5 mg/kg course of rATG. This observation is frequent as shown in the seminal study by Brennan D et al, where only 68.6% of the patients received the intended five doses of rATG (22). We also observed a similar pattern analyzing this strategy in a larger cohort of high-risk kidney transplant recipients (16). The reasons to reduce the intended total dose are primarily safety issues, such as leukopenia, thrombocytopenia and surgical complications (22). The trends towards higher incidence of acute rejection among patients receiving < 5 mg/kg in the rATG-5 group suggests that the inability to complete the intended 5 dose course, as a consequence of impeding toxicity, may increase the risk of acute rejection.

Please define the following: „low HLA compatibility, long cold ischemia time, priority criterion, and donor type”

Low HLA compatibility was defined as more than 3 HLA mismatches.

Long cold ischemia time is defined as cold ischemia time higher than 24 hours

Priority criterion is the priority status attributed to a patient with imminent lack of access for peritoneal hemodialysis during the allocation process

Donor types are living, and standard or expanded criteria deceased donors

This information was added to the method section.

“Altogether, the stable eGFR trajectories over the 36 months, the early and persistent low-grade proteinuria, and the higher cg Banff scores in the rATG-3 group suggest that the initial difference in eGFR was derived from unfavorable donor characteristics and recovery from ischemia reperfusion injury.” – but according to data in Table 1 donor characteristics did not differ significantly between both groups, please clarify.

YES! The editor is correct. In fact, this paragraph is in our discussion as a speculation for the unexpected findings in kidney function. We rule out the role of acute rejection (considering that we were diligent in excluding this diagnosis by performing a higher number of biopsies in this group of patients). We were left with this interaction, “unfavorable donor characteristics and recovery from ischemia reperfusion injury”. We called “unfavorable donor characteristics” a combination of older age (42 vs. 45.5 years, p-0.421), lower male donors (65% vs. 49.1%, p=0.02) and consequent unbalanced donor/recipient match (p=0.043), donor acute kidney injury as measured by Δ creatinine (0.2 vs. 0.5 mg/dl, p=0.019), and longer cold ischemia time (22 vs. 24 hours, p=0.06). We stressed in the discussion that this is speculative.
